# Molecular Epidemiology and Surveillance of Human Adenovirus and Rotavirus A Associated Gastroenteritis in Riyadh, Saudi Arabia

**DOI:** 10.3390/tropicalmed8050279

**Published:** 2023-05-15

**Authors:** Saleh Eifan, Islam Nour, Atif Hanif, Abdulkarim Alhetheel, Ibrahim Al-Ashkar

**Affiliations:** 1 Molecular Virology Laboratory, Department of Botany and Microbiology, College of Science, King Saud University, Riyadh 11451, Saudi Arabia; 2 Virology Laboratory, Department of Pathology and Laboratory Medicine, College of Medicine, King Saud University, Riyadh 11451, Saudi Arabia; 3 Department of Plant Production, College of Food and Agriculture, King Saud University, Riyadh 11451, Saudi Arabia

**Keywords:** human rotavirus A, human adenovirus, gastroenteritis, epidemiology, environmental, genotype 41, G2 lineage

## Abstract

In Saudi Arabia, acute gastroenteritis (GE) is a common illness affecting children and adults; however, the extent to which human rotavirus A (HRV) and human adenovirus (HAdV) strains contribute to the condition is unclear. The surveillance of the GE-causing viruses, HRV and HadV, was performed using polymerase chain reaction, sequencing, and phylogenetic analysis at King Khalid University Hospital. The associations between virus prevalence and meteorological factors were analyzed. The prevalence of HAdV was recorded (7%), followed by HRV (2%). On a gender basis, HAdV infections were found to be dominant in females (5:2) (*U* = 407.5; *p* < 0.0001), whereas HRV was only detected in males (*U* = 50; *p* < 0.0001). A significantly higher HAdV prevalence was recorded at the age of 3.5 ± 0.63 years (21.1%; *p* = 0.00047), whereas HRV cases were found equally distributed between <3 years and 3–5 years. The highest HAdV prevalence was recorded in autumn, followed by winter and spring. A significant correlation was detected between humidity and the total number of recorded cases (*p* = 0.011). Phylogenetic analysis depicted the dominance of HAdV type 41 and the G2 lineage of HRV among circulating strains. The current study uncovered the epidemiology and genotypes of HRV and HadV, and provided forecasting equations for monitoring climatic-mediated outbreaks.

## 1. Introduction

Diarrhea is a common gastroenteritis-related cause of morbidity and mortality in children. Previously, bacterial infections were considered the main cause of gastroenteritis, but due to the developments in viral diagnostics, viruses have been considered the main cause of gastroenteritis over the past 20 years [1]. Gastroenteritis is a major infectious disorder affecting people of all ages globally, particularly young children suffering from acute forms of gastroenteritis. Especially in the developing world, viral infections are reported as a significant source of gastroenteritis [2]. Despite the fact that the majority of gastroenteritis infections are self-limiting, they continue to be major contributors to morbidity and economic losses [3]. Among children under the age of five, diarrhea is a serious public health concern and a potentially infectious disease with high rates of morbidity and mortality. It has been estimated that gastric rotavirus infections cause 197,000 fatalities in children under the age of five, and high incidence places a heavy economic burden on both high- and low-income nations [4]. Around the world, rotaviruses account for 9% of under-five mortality and are a major cause of infantile acute diarrhea (AD) [5]. The rotavirus is a non-enveloped, double-stranded RNA virus with a triple-layered icosahedral capsid that encodes six structural viral proteins and six non-structural viral proteins linked to virulence [6]. Additionally, it has two surface proteins, the G-type (glycosylated VP7) and the P-type (VP4 proteins susceptible to proteases), as described in rotavirus genotyping based on dual classification [7,8]. The most significant cause of acute infantile gastroenteritis, after rotavirus, is adenovirus. Adenoviruses are potential causes of AD in infants and gastroenteritis (GE), accounting for 2–6% of the incidence worldwide [9]. Adenoviruses are non-enveloped icosahedral viruses with a double-stranded linear DNA of 34–36 kb. These include 88 human serotypes grouped into seven human adenovirus species, A–G [10]. Gastrointestinal infections are commonly caused by subgroups A, D, and F. Serotypes 40 and 41 of subgroup F and serotype 31 of subgroup A are mainly associated with GE [11,12]. However, limited studies have been conducted on the detection of circulating HAdV strains in patients in Saudi Arabia [13,14,15].

Therefore, the current study aimed at molecular characterization of circulating HAdV and HRV in the King Khalid University Hospital (KKUH) in Riyadh, Saudi Arabia. Moreover, the hexon- and VP7-derived sequences of the detected HAdV and HRV were sequenced for phylogenetic analysis. Furthermore, seasonal influences were inspected in the context of HRV and HAdV prevalence.

## 2. Materials and Methods

### 2.1. Clinical Samples Collection and Preparation

Fecal samples from patients visiting King Khalid University Hospital (KKUH) in Riyadh because of diarrhea or gastroenteritis symptoms between February 2017 and January 2018 were selected retrospectively. Acute GE was defined as diarrhea and/or vomiting for >7 days, which could be associated with abdominal pain, fever, and anorexia [16]. However, AD is defined as the passage of excessive watery or frequent stools with surged water content [17]. Nosocomial cases and chronic diarrheal cases (lasting for >2 weeks) were excluded from this study. Consequently, 100 fecal samples were included and collected by the addition of 3× volume of DNA/RNA Shield reagent (Zymo Research, Irvine, CA, USA). The fecal samples were suspended in 2 mL of phosphate-buffered saline, incubated for 1 h at 4 °C, and centrifuged at 6000× *g* for 5 min to remove cellular debris and bacteria. This was followed by supernatant filtration using a 0.45 µm syringe filter (Millipore, Billerica, MA, USA). Metrological data, including temperature, humidity, and wind speed, were obtained from AccuWeather Riyadh https://www.accuweather.com/en/sa/riyadh/ on the day of the clinical sample collection.

### 2.2. Nucleic Acid Extraction and PCR Detection of Enteric Viruses

Nucleic acid was extracted from 400 μL of filtered supernatant and eluted in 50 μL of molecular-grade water using the PowerViral^®^ Environmental RNA/DNA Isolation kit (MO BIO Lab, Carlsbad, CA, USA) by following manufacturer instructions. The yielded RNA was initially reverse transcribed using the Sensiscript^®^ RT Kit (SensRT; Qiagen GmbH, Hilden, Germany) in a 20 μL reaction mixture consisting of 2 μL template RNA and 18 μL RT mixture (containing 2 μL 10× RT buffer, 2 μL dNTPs, 2 μL random hexamers [final concentration of 10 μM], 1 μL Sensiscript RT, 1 μL RNase inhibitor [40 U·μL^−1^], and 10 μL nuclease-free water with incubation at 37 °C for 1 h). For HRV detection, PCR was conducted in a 20 μL reaction mixture consisting of 2 μL cDNA template, 500 nM RComb-F: 5′-CCACAAYTDTATTGTGATTA-3′ and RComb-R: 5′-CCCATYGATATCCAYTTATT-3′ [18], 500 nM reverse primer, and 1× Phusion Master Mix (Thermo Fisher Scientific, Waltham, MA, USA). This was performed under the following reaction conditions: 98 °C for 30 s, followed by 40 cycles of 98 °C for 10 s, 50 °C for 30 s, and 72 °C for 30 s each, and a final extension at 72 °C for 5 min. Conversely, HAdV was directly detected in a 20 μL reaction mixture composed of 2 μL DNA template, 300 nM AdFhex-F: 5′-GCCACCGATACCTACTTCAGCCTG-3′ and 300 nM AdFhex-R: 5′-GGCAGTGCCGGAGTAGGGTTTAAA-3′ [19], and 1× Phusion Master Mix under the following reaction conditions: 98 °C for 30 s, followed by 40 cycles of 98 °C for 10 s and 72 °C for 30 s each, and a final extension at 72 °C for 5 min.

### 2.3. Amplicon Purification and Sequencing

To confirm the expected PCR products, gel electrophoresis was applied to 2× agarose concentration. These amplicons were purified using the Wizard^®^ SV Gel and PCR Clean-Up System (Promega Co., Madison, WI, USA) according to the manufacturer’s instructions. Consequently, the cleaned-up amplicons were sequenced using the Applied Biosystem PRISM^®^ 7000 Sequence Detection System (Thermo Fisher Scientific, USA).

### 2.4. Phylogenetic Analysis

Pairwise alignment, comparisons, and visualization of genomes were conducted using BioEdit version 7.2.0 (http://www.mbio.ncsu.edu/BioEdit/page2.html accessed on 2 March 2023). Bootstrapped, neighbor-joining phylogenetic trees with 1000 replicates were constructed using MEGA X [20]. Genetic distances were evaluated using the best-fitting substitution model. For HRV phylogenetic analysis, the HRV sequences of the present study were compared with the closest sequences upon blasting, in addition to the HRV G1 lineage represented by G1P [8] and G9 lineage including G9P [8], since the latter genotypes were recorded with high prevalence in Saudi Arabia and surrounding countries [21,22,23]. The G12 lineage was used as the outgroup.

### 2.5. Statistical Analysis

Normally distributed continuous data were displayed as mean $\left(\overset{-}{x}\right)$ ±  standard deviation and were analyzed using Pearson’s correlation as convenient. Non-parametric data were analyzed using the Mann–Whitney *U* test. Categorical data were presented as frequencies and were analyzed using the χ^2^ test. Moreover, multivariate logistic regression analysis was conducted to define the independent risk factors. *p*  <  0.05 was regarded as statistically significant. The relationships between both viruses (as dependent variables) and meteorological factors (as independent variables) were fitted using linear curve fitting. Statistical analyses were conducted using the XL-STAT statistical package software (Ver. 2019, Excel Add-ins soft SARL, New York, NY, USA).

## 3. Results

### 3.1. Clinical Demographics

We collected stool samples from 100 patients, including 52 males and 48 females (male:female, 1.083:1), from February 2017 to January 2018. The patients were aged between 2 months and 68 years.

### 3.2. Gender- and Age-Based Distribution of HRV and HAdV

Overall, HAdV had the highest prevalence (7%), followed by HRV (2%). On a gender basis, HAdV infections were more dominant in females than in males (5:2) (*U* = 407.5; *p* < 0.0001), whereas HRV was mainly prevalent in males (*U* = 50; *p* < 0.0001). On the other hand, the age-based distribution showed that most patients were 3–5 years old (4/19; 21.1%; $\left(\overset{-}{x}\right)$ = 3.5 ± 0.6), followed by under 3 years old (2/42; 4.76%; $\left(\overset{-}{x}\right)$ = 2 months) in HAdV cases (χ^2^ = 22.23; *p* = 0.00047). Strikingly, one HAdV case was recorded in a 54-year-old patient. In HRV cases, cases were equally distributed in under 3-year and 3- to 5-year-old groups (1/2; 50%; $\left(\overset{-}{x}\right)$ = 3.5 ± 2.12) with a male dominance (Table 1). Moreover, in patients aged ≤5 years, an HRV prevalence of 3.27% (2/61) was recorded. Furthermore, HRV infections were absent in children above 5 years old and adults. However, there was an insignificant association between HRV prevalence and age (χ^2^ = 1.859; *p* > 0.05).

### 3.3. Temporal Distribution of HAdV and HRV

**Figure 1:**
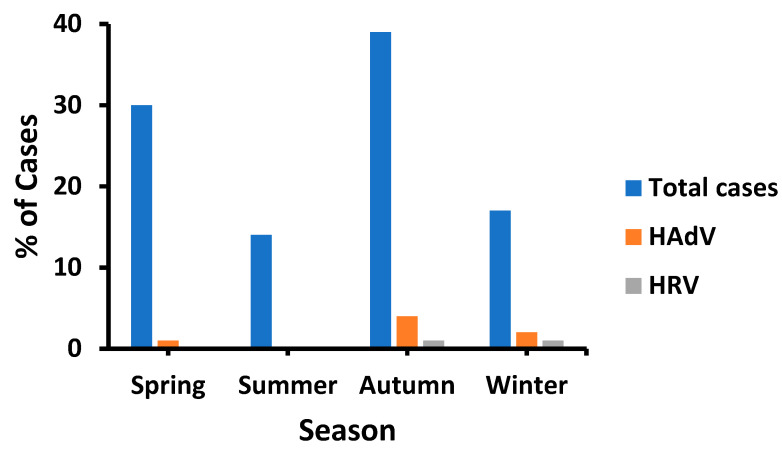
Seasonal prevalence of HAdV and HRV cases in patients between February 2017 and January 2018.

**Figure 2:**
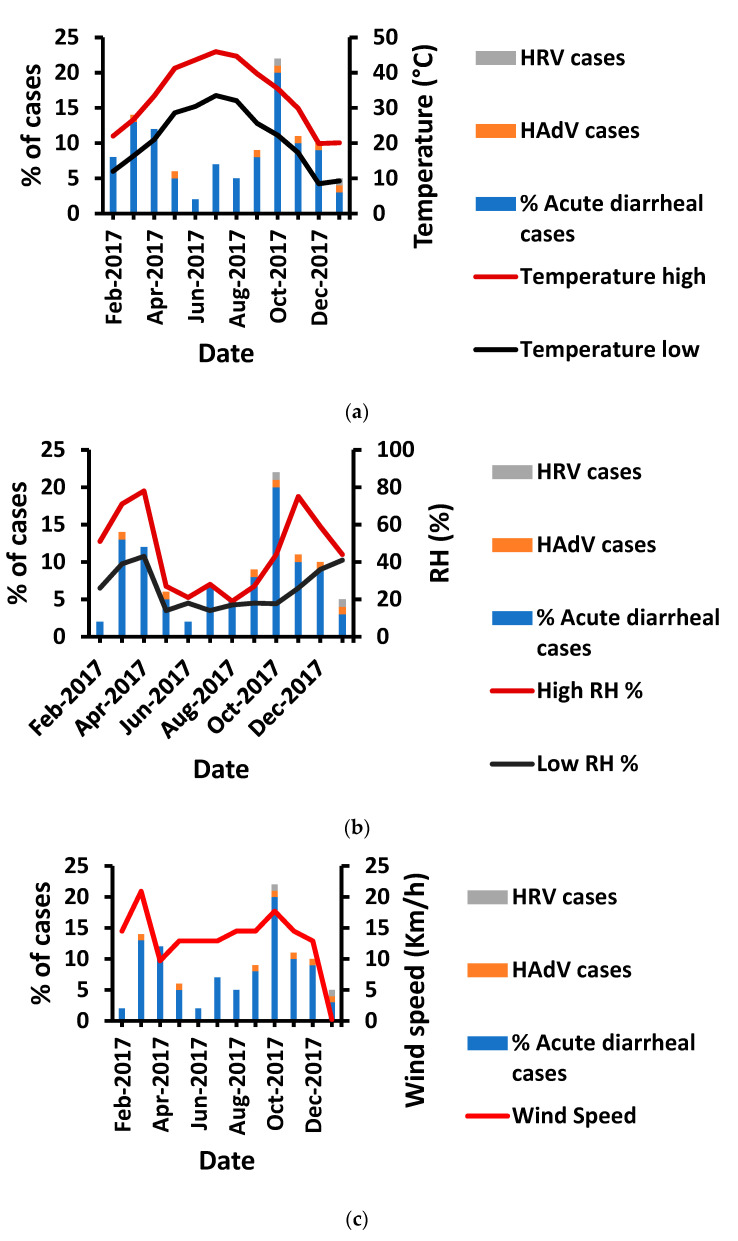
Environmental influences, involving (**a**) temperature, (**b**) humidity, and (**c**) wind speed, on HRV and HAdV prevalence and total number of recorded cases.

HAdV had a higher prevalence in autumn and early winter, followed by spring; however, there were no cases in summer (Figure 1). Likewise, the recorded cases were the lowest (14%) in summer, unlike in autumn (39%). HRV infection occurred equally in autumn and winter. Notably, both HRV and HAdV were detected in January, although fewer diarrheal cases were recorded during this month (Figure 2). Such distribution patterns mandated the study of environmental (meteorological) influences on the prevalence of HRV and HAdV.

### 3.4. Temperature Impact on HAdV and HRV Prevalence

The recorded cases were the highest at moderate temperatures (high: 35 °C, low: 22 °C). The lowest prevalence generally occurred in summer at high temperatures (high: 43.6 °C–45.9 °C, low: 30.4 °C–33.5 °C), particularly in June (Figure 2a). Likewise, the HAdV prevalence displayed the same pattern. However, HRV favored even lower temperature ranges (high: 20 °C–35 °C, low: 9 °C–22 °C). Despite the observed pattern, temperature had an insignificant influence on the prevalence of both viruses (*p* > 0.05) (Table 2).

### 3.5. Humidity Impact on HAdV and HRV Prevalence

HRV cases were only detected at a moderate relative humidity range (high: 41–44, low: 17.7–41; *p* = 0.502) (Figure 2b). However, HAdV cases were equally distributed across all relative humidity ranges from low (high: 27%, low: 14%) to moderate (high: 44%, low: 17.7%) to high relative humidity (high: 75%, low: 26%) (*p* = 0.404). Consequently, HRV and HAdV were insignificantly affected by relative humidity (*p* > 0.05). However, the total number of cases was significantly correlated with relative humidity (*p* = 0.011) (Table 2).

### 3.6. Influence of Wind Speed on HRV and HAdV Prevalence

HAdV infections were significantly associated with recorded diarrheal cases (*p* = 0.018) and occurred at all ranges of wind speed, including low (0.24 Km/h), moderate (12.9 Km/h), and high (17.7–20.9 Km/h) (Figure 2c). Moreover, HRV was detected at both high (17.7 Km/h) and low wind speeds (0.24 Km/h). Therefore, there was an insignificant influence of wind speed on HRV and HAdV prevalence (*p* = 0.77 and 0.796, respectively) (Table 2).

### 3.7. Prevalence of G2 Lineage of HRV in Patients

**Figure 3:**
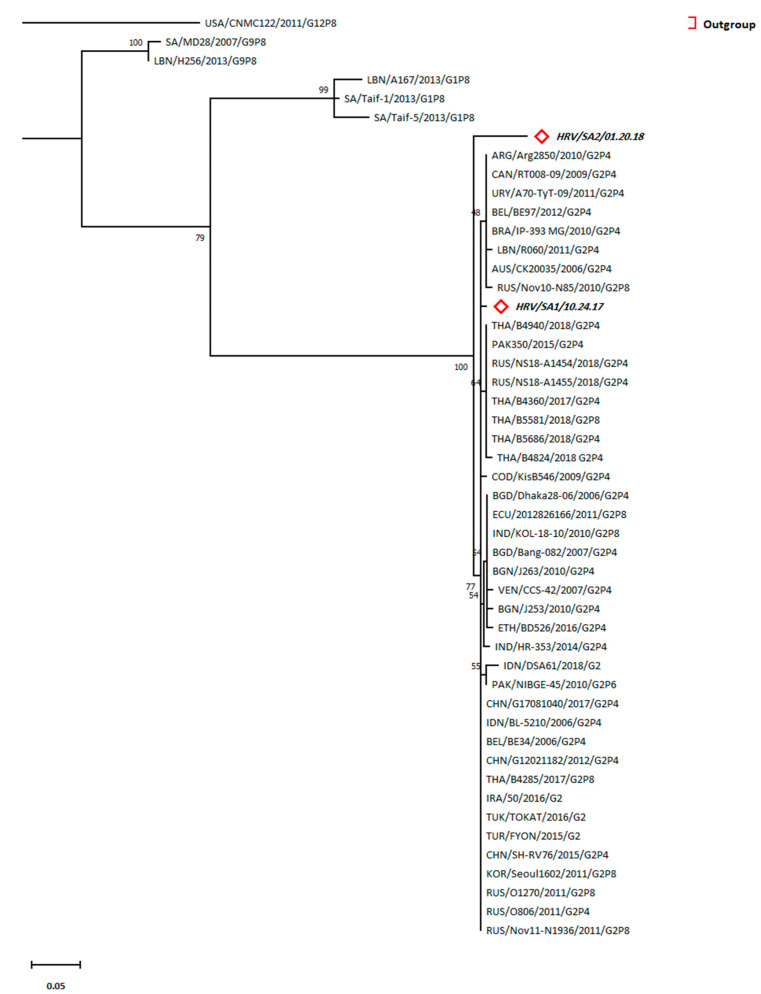
Phylogenetic tree for the VP7-derived sequences of HRVA generated by maximum likelihood method and Tamura 3-parameter model. 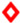 Refers to HRV sequences (bold italic) belonging to this study.

Phylogenetic analysis showed a consistent relationship between the two HRV sequences (SAU/SA-01/10.24.17 and SAU/SA-02/1.20.18) and the lineage G2 rather than lineage G1 and G9 (Figure 3). Pairwise distancing showed the highest closeness of SAU/SA-01/10.24.17 (*d* = 0.0058) to sequences from Iraq (IRA/50/2016/G2), Turkey (TUR/TOKAT/2016/G2 and TUR/FYON/2015/G2), China (CHN/G17081040/2017/G2P4, CHN/G12021182/2012/G2P4, and CHN/SH-RV76/2015/G2P4), Russia (RUS/O806/2011/G2P4, RUS/O1270/2011/G2P8, and RUS/Nov11-N1936/2011/G2P8), South Korea (KOR/Seoul1602/2011/G2P8), Thailand (THA/B4285/2017/G2P8), Belgium (BEL/BE34/2006/G2P4), and Indonesia (IDN/BL-5210/2006/G2P4). Moreover, the other HRV sequence (SAU/SA-02/1.20.18) was closer to the same above sequences (*d* = 0.054) than to the SAU/SA-01/10.24.17 detected in the patients (*d* = 0.06) (Table S1). Remarkably, Saudi Arabian sequences belonging to the G1 lineage, in particular SAU/Taif-1/2013/G1P8 (*d* = 0.235 and 0.26), were more related to our sequences than to the Lebanese sequence LBN/A167/2013/G1P8 (*d* = 0.3096 and 0.287).

The accession numbers of the sequences used in the phylogenetic analysis of HRV, sequence nominations, and abbreviations are included in Table S2. We describe the tree with the greatest log-likelihood (−732.53). The Neighbor-Join and BioNJ algorithms were applied to a matrix of pairwise distances for primary tree generation comparison and top log-likelihood topology selection. Some sites were considered evolutionarily invariable ([+*I*], 51.65% sites) according to the substitution model selection (Table S3). The branch lengths were estimated using the substitutions/site count for tree scaling. A total of 173 positions existed in the final dataset.

### 3.8. Predominance of HAdV Type 41

**Figure 4:**
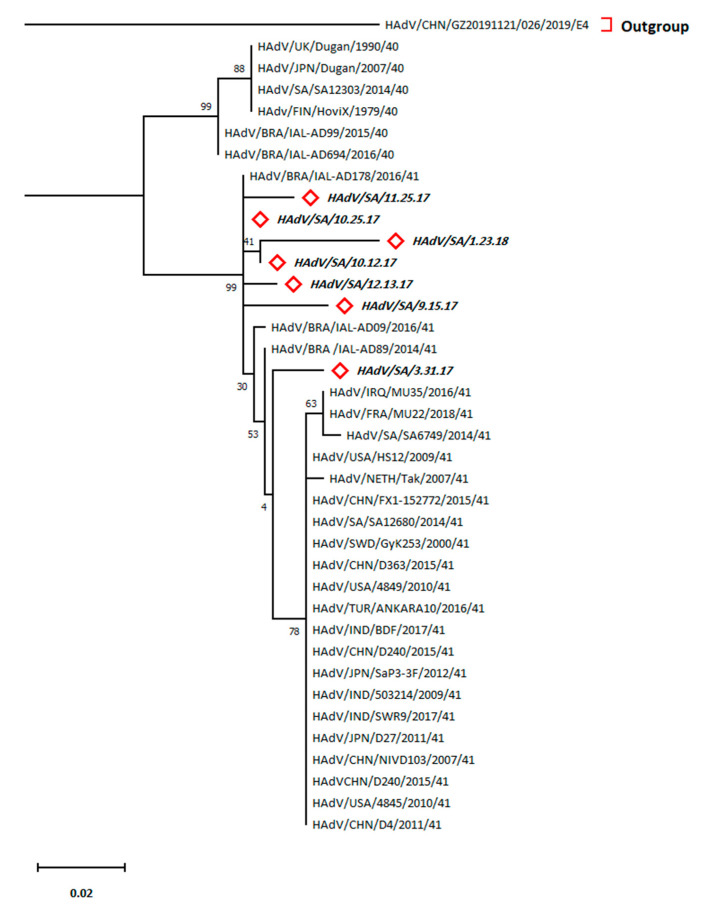
Phylogenetic tree for the HAdV hexon sequences constructed using maximum likelihood method and Jukes–Cantor model. 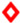 Denotes HAdV sequences (bold italic) belonging to this study.

The phylogenetic analysis depicted a typical relationship of these hexon sequences of HAdV to serotype 41 of F species rather than serotype 40 of HAdV (type F) (Figure 4). Pairwise distancing revealed that the Brazilian sequence (HAdV/BRA/IAL-AD178/2016/41) was similar to HAdV/SAU/10.25.17 (*d* = 0) (Table S4). Moreover, the latter sequence was close to three other Brazilian sequences (BRA/IAL-AD178/2016/41, BRA /IAL-AD89/2014/41, and BRA/IAL-AD09/2016/41) and the HAdV/SAU/10.25.17 sequence (*d* = 0.00384). Additionally, the Brazilian strain (HAdV/BRA/IAL-AD99/2015/40) was the closest sequence among serotype 41 to the HAdV/SAU/10.25.17 sequence (*d* = 0.0393) in the present study.

The accession numbers of the sequences used in the phylogenetic analysis of HAdV, sequence nominations, and abbreviations are included in Table S5. The highest log-likelihood tree (−733.50) is displayed. The percentage of the clustered taxa-based trees is provided next to the branches. Both the Neighbor-Join and BioNJ algorithms were applied to the maximum composite likelihood-mediated pairwise distance matrix to yield the initial tree(s) for the heuristic search followed by higher log-likelihood topology selection. The Jukes–Cantor substitution model was used based on the best-fitting model selection for phylogenetic tree generation (Table S6). The tree is drawn to scale, with branch lengths measured as the number of substitutions per site. The horizontal distance linking two hexon sequences is proportional to the genetic distances between the inter-hexon sequences. There were a total of 261 positions in the final dataset.

## 4. Discussion

Diarrhea is a potential health issue globally and is considered the fourth leading cause of under-5 mortalities [24]. We found a higher dominance of diarrheal cases associated with viral infections (9%), which is lower than that previously reported in Saudi Arabia (21%) [25] and Qatar (48%) [26]. This discrepancy could be because of the spatial and temporal variations, sample size differences, and overwhelming infantile contributions. However, a closer or even lower virus-mediated diarrheal prevalence of approximately 7% post-vaccination program implementation was recorded in Andhra Pradesh, India [27]. Likewise, the Saudi national immunization program was reported to decrease viral-mediated GE from 31% in 2012 to 3.9% in 2015, which concurs with the further decline in GE depicted in our study [28].

On the other hand, the majority of HRV cases were males, which is in agreement with a previous study [28]. However, HAdV was more prevalent in females than in males, contrary to those reported elsewhere [29]; this could be because of geographical differences. Moreover, according to other studies, the prevalence of HAdV and HRV was high in children younger than 5 years [30,31] which supports our findings. Despite the higher prevalence of HRV in this age group, an insignificant difference was noted, s depicted in previous results [32]. A decade after Taiwan’s introduction of the rotavirus vaccination, children under the age of five who were admitted with acute gastroenteritis to 10 hospitals in Taiwan depicted an 8.7% rotavirus detection rate [33]. Similarly to our findings, studies in Belgium and Lebanon reported a rotavirus prevalence of 6.4% and 13.6%, respectively [34,35]. In Japan, the prevalence of HRV was recorded between 2014 and 2020 in patients with acute gastroenteritis after the initiation of the vaccine program. The rates of HRV detection dropped from 44.7% (2014–2015) to 35.4% (2018–2019); however, no HRV cases were detected in the samples collected during 2019–2020 [36].

However, these studies described a significantly higher HRV prevalence than that in the present study, which is due to their restricted study of ≤5-year-old patients. Furthermore, the rotavirus vaccination was added to the National Immunization Program in Saudi Arabia in 2013 with a two-dose schedule at the ages of 2 and 4 months [37], and lower HRV prevalence may be attributed to the efficacy of the vaccination program. Similarly, in the United States during the post-vaccination period (2007–2018), the median annual percentage of rotavirus tests that were positive decreased from 25.6% (range: 25.2–29.4%) to 6.1% (range: 2.6–11.1%) between the pre-vaccine and post-vaccine periods [38]. Moreover, in Finland, during the post-vaccination period (2008–2018), a very low level of rotavirus incidence (<5/10,000 children) was reported [39].

The seasonal impact on HAdV and HRV prevalence has been intensively studied; however, various findings have been obtained. Higher HRV prevalence was reported in winter (*n* = 4) in Lebanon [35], China [40], India [27], and Eastern Mediterranean region (*n* = 1) [41]; autumn and winter (*n* = 1) in Saudi Arabia [42]; late autumn together with winter and early spring (*n* = 1) in Bangladesh [30]; summer (*n* = 2) in Egypt and Yemen [43,44]; and in all seasons (*n* = 1) in Pakistan [32]. In our study, HRV was detected in autumn and winter, similar to that reported in Saudi Arabia [28]. However, HAdV had the highest prevalence in winter (*n* = 1) in Italy [45], autumn (*n* = 1) in Iran [46], and spring (*n* = 1) in Saudi Arabia [13]. We found a higher HAdV prevalence in autumn, unlike that depicted earlier in Saudi Arabia [13]. The shift in the HAdV prevalence pattern could be justified by temporal differences. Likewise, a 20-year retrospective surveillance conducted in Switzerland uncovered a seasonal shift of notable HAdV peaks from spring in 1998 to summer in 2009 to winter in 2010 [47].

Moreover, a higher HAdV prevalence was detected at lower temperatures in autumn and winter, which agrees with previous findings [28]. However, Xie et al. recorded a higher HAdV prevalence in summer in China because of spatial differences [48]. Moreover, HRV was predominant at lower temperatures, as reported previously [23]. The present study showed moderate humidity as the favorable condition for HRV, whereas HAdV had the same prevalence at all humidity levels. However, Arowolo et al. noted that HAdV prevalence was greater in dry environments with low humidity [49]. Similarly, HRV was found to be more prevalent in the dry season with low temperature and lower humidity, which disagrees with our outcomes [49,50]. This contradiction is due to the climatic differences (the latter study was conducted in a tropical climate). Furthermore, low temperature and low humidity may enhance the survival of enteric viruses in pediatric GE [51]. Additionally, a previous study identified a correlation between wind speed and peak viral activity [52]. On the other hand, an insignificant impact of wind speed on both HRV and HAdV was observed in our study. The observational difference is due to the different viruses inspected in the previous study, as well as the geographical preferences. In addition, wind speed did not influence viral seasonality elsewhere, which agrees with the present finding [53].

On the other hand, phylogenetic analysis of HRV showed that it was entirely clustered in lineage G2. However, G1 was frequently reported as the prevalent lineage of HRV, as reported by several studies in Saudi Arabia, Lebanon, and Bahrain [22,23,54]. G3 was the most prevalent genotype in Qatar [55]. A recent study highlighted a surge in G2 incidence, particularly the G2P [4] genotype, notably after vaccination, which could partially justify our results [21]. Thus, vaccination-mediated selection pressure may have favored G2 rather than G1 of HRVA. However, the discrepancy in the Qatar findings could be due to the expats’ contribution, comprising over 80% of the community structure, which might have resulted in the supremacy of the G3 genotype [56]. Moreover, HAdV phylogenetic analysis revealed that the HAdV serotype 41 was prevalent in all detected patients. HAdV types 40 and 41 were detected in diarrheal patients from Riyadh, Jeddah, and Mecca in Saudi Arabia, which agrees with our observations of our detected sequences being clustered along with HAdV type 41 [13]. Species F is usually associated with acute GE in children [57]. Eventually, the current study was limited by the number of diarrheal cases admitted to KKUH during a one-year period (i.e., the sample size was too small), which might not be adequate to correlate the rates of positive cases with factors, such as gender and temporal factors. Moreover, other infectious agents could have attributed to the incidence of these cases. However, our study intended to detect viral gastroenteritis cases, particularly HAdV- and HRV-related cases. In conclusion, the present study uncovered the HRV and HAdV types and their epidemiology among hospitalized patients in KKUH from February 2017 to January 2018. Additionally, type analysis is necessary when realizing any imported new strains that could affect vaccination efficacy, as in the case of HRV, or result in different diseases, as in the case of HAdV. Our study of meteorological factors’ influences on the prevalence of both viruses provided prediction equations, which are useful for monitoring the incidence of climatic-mediated outbreaks. It is recommended that in future studies, multiple hospitals and health care settings should be included and a larger sample size including different age group subjects should be investigated. Moreover, other enteric viruses causing gastroenteritis and acute diarrhea, such as Norvovirus, Astrovirus, hepatitis A virus, hepatitis E virus and Sapovirus, including environmental factors associated with the disease, should be analyzed for the assessment of possible occurrence patterns or attributing factors for future outbreaks.

## Figures and Tables

**Table 1:** Gender and age distribution of patients with HAdV and HRV infections.

Age (Years)	HRV Cases			HAdV Cases		
	Male	Female	Total (%)	Male	Female	Total (%)
<3	1/27 (3.7%)	0/15 (0%)	1%	1/27 (3.7%)	1/15 (6.7%)	2%
3–5	1/9 (11.1%)	0/10 (0%)	1%	0/9 (0%)	4/10 (40%)	4%
6–12	0/13 (0%)	0/19 (0%)	0%	0/13 (0%)	0/19 (0%)	0%
13–18	0/1 (0%)	0/3 (0%)	0%	0/1 (0%)	0/3 (0%)	0%
19–60	0/1 (0%)	0	0%	1/1 (100%)	0	1%
60+	0/1 (0%)	0/1 (0%)	0%	0/1 (0%)	0/1 (0%)	0%
Total (%)	2/52 (3.8%)	0/48 (0%)	2%	2/52 (3.8%)	5/48 (10.4%)	7%

**Table 2:** Significance of the influences of environmental factors on the prevalence of both viruses and associated diseases.

Environmental Factor	Virus and Associated Diseases	*R* ^2^	RMSE	Equation **
High temperature (T_H_)	HAdV	0.449	0.469	*Prev*_HAdV_ = 0.34 + 3.3 × 10^−2^ × T_H_
	HRV	0.002	0.534	*Prev*_HRV_ = 0.32 − 1.76 × 10^−3^ × T_H_
Low temperature (T_L_)	HAdV	0.099	1.2	*Prev*_HAdV_ = 1.52 − 3.46 × 10^−2^ × T_L_
	HRV	0.134	0.497	*Prev*_HRV_ = 0.54 − 1.7 × 10^−2^ × T_L_
Relative humidity (RH%)	HAdV	0.140	0.830	*Prev*_HAdV_ = 1.53 − 1.4 × 10^−2^ × RH%
	HRV	0.216	0.473	*Prev*_HRV_ = 0.68 − 1.04 × 10^−2^ × RH%
Wind speed (WS)	HAdV	0.079	1.051	*Prev*_HAdV_ = 1.36 − 5.27 × 10^−2^ × WS
	HRV	0.47	0.389	*Prev*_HRV_ = 0.72 − 6.27 × 10^−2^ × WS

*Prev* denotes the prevalence of the virus. ** These equations, resulting from metrological data analysis, can be used for future prediction of HAdV and HRV incidences, relying on provided environmental data.

## Data Availability

Sequences used in the present study for phylogenetic analysis are available at the NCBI GenBank repository with accession numbers mentioned in Tables S2 and S5.

## Supplementary Materials

The following supporting information can be downloaded at: https://www.mdpi.com/article/10.3390/tropicalmed8050279/s1, Figure S1: HRV primers positions; Table S1: Evolutionary divergence estimates between Sequences of HRV; Table S2: Sequences used for phylogenetic analysis of HRV; Table S3: Best fitting model selection for HRV using Maximum Likelihood fits of 24 different nucleotide substitution models; Table S4: Evolutionary divergence estimates between Sequences of HAdV; Table S5: Sequences used for phylogenetic analysis of HAdV; Table S6: Best fitting model selection for HAdV using Maximum Likelihood fits of 24 different nucleotide substitution models; FAS file 1 HRV 94 sequences; FAS file 2 HRV 94 sequences alignment.

## References

[1] Malik Y.S., Verma A.K., Kumar N., Touil N., Karthik K., Tiwari R., Bora D.P., Dhama K., Ghosh S., Hemida M.G. (2019). Advances in diagnostic approaches for viral etiologies of diarrhea: From the lab to the field. Front. Microbiol..

[2] Tate J.E., Burton A.H., Boschi-Pinto C., Parashar U.D., Agocs M., Serhan F., de Oliveira L., Mwenda J.M., Mihigo R., World Health Organization–Coordinated Global Rotavirus Surveillance Network (2016). Global, regional, and national estimates of rotavirus mortality in children < 5 years of age, 2000–2013. Clin. Infect. Dis..

[3] Hoffman S., Maculloch B., Batz M. (2015). Economic burden of major foodborne illnesses acquired in the United States (No. 1476-2016–120935). Econ. Bull..

[4] Lanata C.F., Fischer-Walker C.L., Olascoaga A.C., Torres C.X., Aryee M.J., Black R.E., Child Health Epidemiology Reference Group of the World Health Organization and UNICEF (2013). Global causes of diarrheal disease mortality in children < 5 years of age: A systematic review. PLoS ONE.

[5] Goel A.K., Chawla S., Dhingra A., Thiyagarajan V., Nair N.P. (2021). Rotavirus Diarrhea and Its Determinants among Under-Five Children Admitted in a Tertiary Care Hospital of Southern Haryana, India. Indian J. Pediatr..

[6] Desselberger U. (2014). Rotaviruses. Virus Res..

[7] Nour I., Hanif A., Ryan M., Eifan S. (2021). Insights into Gastrointestinal Virome: Etiology and Public Exposure. Water.

[8] O’Ryan M. (2009). The Ever-Changing Landscape of Rotavirus Serotypes. Pediatr. Infect. Dis. J..

[9] Mohammadi J., Amini R., Akbari A., Amraei M., Mahmoudvand S., Jalilian F.A. (2020). Prevalence and Seasonal Frequency of Acute Viral Gastroenteritis in Children Less than 5 Years in Ilam, Iran. Prevalence..

[10] Dhingra A., Hage E., Ganzenmueller T., Böttcher S., Hofmann J., Hamprecht K., Obermeier P., Rath B., Hausmann F., Dobner T. (2019). Molecular Evolution of Human Adenovirus (HAdV) Species C. Sci. Rep..

[11] Lee B., Damon C.F., Platts-Mills J.A. (2020). Pediatric acute gastroenteritis due to adenovirus 40/41 in low-and middle-income countries. Curr. Opin. Infect. Dis..

[12] Lion T. (2019). Adenovirus persistence, reactivation, and clinical management. FEBS Lett..

[13] Tayeb H.T., Dela Cruz D.M., Al-Qahtani A., Al-Ahdal M.N., Carter M.J. (2008). Enteric Viruses in Pediatric Diarrhea in Saudi Arabia. J. Med. Virol..

[14] Meqdam M.M., Thwiny I.R. (2007). Prevalence of Group a Rotavirus, Enteric Adenovirus, Norovirus and Astrovirus Infections among Children with Acute Gastroenteritis in Al-Qassim, Saudi Arabia. Pak. J. Med. Sci..

[15] Akhtar J., Qadri S.M.H., Myint S.H. (1995). Gastrointestinal Adenovirus Infections in a Tertiary Referral Centre in Saudi Arabia. Eur. J. Clin. Microbiol. Infect. Dis..

[16] Sdiri-Loulizi K., Gharbi-Khélifi H., de Rougemont A., Chouchane S., Sakly N., Ambert-Balay K., Hassine M., Guédiche M.N., Aouni M., Pothier P. (2008). Acute Infantile Gastroenteritis Associated with Human Enteric Viruses in Tunisia. J. Clin. Microbiol..

[17] Ahmad S.A.M., Morsy A.T.A. (2022). Pathogens diarrhea in children, risks and treatment. J. Egypt. Soc. Parasitol..

[18] Nour I., Hanif A., Alanazi I.O., Al-Ashkar I., Alhetheel A., Eifan S. (2021). Novel insights of waterborne human rotavirus A in Riyadh (Saudi Arabia) involving G2 predominance and emergence of a thermotolerant sequence. Sci. Rep..

[19] Dey R.S., Ghosh S., Chawla-Sarkar M., Panchalingam S., Nataro J.P., Sur D., Manna B., Ramamurthy T. (2011). Circulation of a Novel Pattern of Infections by Enteric Adenovirus Serotype 41 among Children below 5 Years of Age in Kolkata, India. J. Clin. Microbiol..

[20] Kumar S., Stecher G., Li M., Knyaz C., Tamura K. (2018). MEGA X: Molecular Evolutionary Genetics Analysis across Computing Platforms. Mol. Biol. Evol..

[21] Almalki S.S. (2018). Circulating Rotavirus G and P Strains Post Rotavirus Vaccination in Eastern Mediterranean Region. Saudi Med. J..

[22] Ali Z., Harastani H., Hammadi M., Reslan L., Ghanem S., Hajar F., Sabra A., Haidar A., Inati A., Rajab M. (2016). Rotavirus Genotypes and Vaccine Effectiveness from a Sentinel, Hospital-Based, Surveillance Study for Three Consecutive Rotavirus Seasons in Lebanon. PLoS ONE.

[23] Aly M., Al Khairy A., Al Johani S., Balkhy H. (2015). Unusual Rotavirus Genotypes among Children with Acute Diarrhea in Saudi Arabia. BMC Infect. Dis..

[24] Wang H., Naghavi M., Allen C., Barber R.M., Carter A., Casey D.C., Charlson F.J., Chen A.Z., Coates M.M., Coggeshall M. (2016). Global, Regional, and National Life Expectancy, All-Cause Mortality, and Cause-Specific Mortality for 249 Causes of Death, 1980â 2015: A Systematic Analysis for the Global Burden of Disease Study 2015. Lancet.

[25] Al Tabbal A.O., Al Humedi S.S. (2017). Surveillance of the Most Prevalent Medical Diseases among Pediatric Age Groups and Evaluation of the Control Measures Used At Tabuk Hospitals, Saudi Arabia. Open Access Maced. J. Med. Sci..

[26] Abdel-Rahman M.E., Mathew S., Al Thani A.A., Ansari K.A., Yassine H.M. (2021). Clinical Manifestations Associated with Acute Viral Gastroenteritis Pathogens among Pediatric Patients in Qatar. J. Med. Virol..

[27] Badur M., Pidugu V.K.R., Kasala L., Thiyagarajan V. (2021). Acute Gastroenteritis in Children below 5 Years of Age at Tirupati, Andhra Pradesh, India Post Introduction of Rotavirus Vaccine into National Immunization Programme. Indian J. Pediatr..

[28] Hegazi M.A., Sayed M.H., Sindi H.H., Bekhit O.E., El-Deek B.S., Alshoudri F.M.Y., Noorelahi A.K. (2017). Is Rotavirus Still a Major Cause for Diarrheal Illness in Hospitalized Pediatric Patients after Rotavirus Vaccine Introduction in the Saudi National Immunization Program?. Medicine.

[29] Jaff D.O., Aziz T.A., Smith N.R. (2015). The Incidence of Rotavirus and Adenovirus Infections among Children with Diarrhea in Sulaimani Province, Iraq. J. Biosci. Med..

[30] Sharif N., Parvez A.K., Haque A., Talukder A.A., Ushijima H., Dey S.K. (2020). Molecular and Epidemiological Trends of Human Bocavirus and Adenovirus in Children with Acute Gastroenteritis in Bangladesh during 2015 to 2019. J. Med. Virol..

[31] Satter S.M., Aliabadi N., Gastañaduy P.A., Haque W., Mamun A., Flora M.S., Zaman K., Rahman M., Heffelfinger J.D., Luby S.P. (2018). An Update from Hospital-Based Surveillance for Rotavirus Gastroenteritis among Young Children in Bangladesh, July 2012 to June 2017. Vaccine.

[32] Alam M.M., Khurshid A., Shaukat S., Suleman R.M., Sharif S., Angez M., Malik S.A., Ahmed T.M., Aamir U.B., Naeem M. (2013). Epidemiology and Genetic Diversity of Rotavirus Strains in Children with Acute Gastroenteritis in Lahore, Pakistan. PLoS ONE.

[33] Lin F.J., Huang Y.C., Huang Y.C., Huang L.M., Liu C.C., Chi H., Lin H.C., Ho Y.H., Wu F.T., Mu J.J. (2022). Clinical and epidemiological features in hospitalized young children with acute gastroenteritis in Taiwan: A multicentered surveillance through 2014-2017. J. Formos. Med. Assoc..

[34] Zeller M., Rahman M., Heylen E., De Coster S., De Vos S., Arijs I., Novo L., Verstappen N., Van Ranst M., Matthijnssens J. (2010). Rotavirus incidence and genotype distribution before and after national rotavirus vaccine introduction in Belgium. Vaccine..

[35] Salami A., Fakih H., Chakkour M., Salloum L., Bahmad H.F., Ghssein G. (2019). Prevalence, Risk Factors and Seasonal Variations of Different Enteropathogens in Lebanese Hospitalized Children with Acute Gastroenteritis. BMC Pediatr..

[36] Okitsu S., Khamrin P., Hikita T., Thongprachum A., Pham N.T., Hoque S.A., Hayakawa S., Maneekarn N., Ushijima H. (2022). Changing distribution of rotavirus A genotypes circulating in Japanese children with acute gastroenteritis in outpatient clinic, 2014–2020. J. Infect. Public Health.

[37] Al-Ayed M.S.Z., Asaad A.M., Qureshi M.A., Hawan A.A. (2017). Epidemiology of group A rotavirus infection after the introduction of monovalent vaccine in the National Immunization Program of Saudi Arabia. J. Med. Virol..

[38] Hallowell B.D., Parashar U.D., Curns A., DeGroote N.P., Tate J.E. (2019). Trends in the laboratory detection of rotavirus before and after implementation of routine rotavirus vaccination—United States, 2000–2018. Morb. Mortal. Wkly. Rep..

[39] Hemming-Harlo M., Gylling A., Herse F., Haavisto I., Nuutinen M., Pasternack M., Kanibir M.N., Hartwig S., Carias C. (2022). Long-term surveillance of rotavirus vaccination after implementation of a national immunization program in Finland (2008–2018). Vaccine.

[40] Kuang X., Gong X., Zhang X., Pan H., Teng Z. (2020). Genetic Diversity of Group A Rotavirus in Acute Gastroenteritis Outpatients in Shanghai from 2017 to 2018. BMC Infect. Dis..

[41] Badur S., Öztürk S., Pereira P., AbdelGhany M., Khalaf M., Lagoubi Y., Ozudogru O., Hanif K., Saha D. (2019). Systematic Review of the Rotavirus Infection Burden in the WHO-EMRO Region. Hum. Vaccines Immunother..

[42] Zaki A., Abousekkien M., Alkholy U.M., Eid A. (2017). Effectiveness and Impact of Rotavirus Vaccines in Saudi Arabia: A Single Hospital-Based Study. Arab J. Gastroenterol..

[43] Wierzba T.F., Abdel-Messih I.A., Abu-Elyazeed R., Putnam S.D., Kamal K.A., Rozmajzl P., Ahmed S.F., Fatah A., Zabedy K., Shaheen H.I. (2006). Clinic-Based Surveillance for Bacterial-and Rotavirus-Associated Diarrhea in Egyptian Children. Am. J. Trop. Med. Hyg..

[44] Al-Badani A., Al-Areqi L., Majily A., Al-Sallami S., Al-Madhagi A., Amood AL-Kamarany M. (2014). Rotavirus Diarrhea among Children in Taiz, Yemen: Prevalence—Risk Factors and Detection of Genotypes. Int. J. Pediatr..

[45] Biscaro V., Piccinelli G., Gargiulo F., Ianiro G., Caruso A., Caccuri F., De Francesco M.A. (2018). Detection and Molecular Characterization of Enteric Viruses in Children with Acute Gastroenteritis in Northern Italy. Infect. Genet. Evol..

[46] Nasab S.D.M., Zali F., Kaghazian H., Aghasadeghi M.R., Mardani R., Gachkar L., Vasmehjani A.A., Ahmadi N., Ghasemzadeh A. (2020). Prevalence of Astrovirus, Adenovirus, and Sapovirus Infections among Iranian Children with Acute Gastroenteritis. Gastroenterol. Hepatol. Bed Bench.

[47] Akello J.O., Kamgang R., Barbani M.T., Suter-Riniker F., Leib S.L., Ramette A. (2020). Epidemiology of Human Adenoviruses: A 20-Year Retrospective Observational Study in Hospitalized Patients in Bern, Switzerland. Clin. Epidemiol..

[48] Xie L., Zhang B., Xiao N., Zhang F., Zhao X., Liu Q., Xie Z., Gao H., Duan Z., Zhong L. (2019). Epidemiology of Human Adenovirus Infection in Children Hospitalized with Lower Respiratory Tract Infections in Hunan, China. J. Med. Virol..

[49] Arowolo K.O., Ayolabi C.I., Lapinski B., Santos J.S., Raboni S.M. (2019). Epidemiology of Enteric Viruses in Children with Gastroenteritis in Ogun State, Nigeria. J. Med. Virol..

[50] D’souza R.M., Hall G., Becker N.G. (2008). 2008. Climatic factors associated with hospitalizations for rotavirus diarrhoea in children under 5 years of age. Epidemiol. Infect..

[51] Chong K.C., Chan E.Y.Y., Lee T.C., Kwok K.L., Lau S.Y.F., Wang P., Lam H.C.Y., Goggins W.B., Mohammad K.N., Leung S.Y. (2021). A 21-Year Retrospective Analysis of Environmental Impacts on Paediatric Acute Gastroenteritis in an Affluent Setting. Sci. Total Environ..

[52] Lim Y.K., Kweon O.J., Kim H.R., Kim T.-H., Lee M.-K. (2020). Clinical Features, Epidemiology, and Climatic Impact of Genotype-Specific Human Metapneumovirus Infections: Long-Term Surveillance of Hospitalized Patients in South Korea. Clin. Infect. Dis..

[53] Price R.H.M., Graham C., Ramalingam S. (2019). Association between Viral Seasonality and Meteorological Factors. Sci. Rep..

[54] Al Musawi M., Zainaldeen H., Shafi F., Anis S., DeAntonio R. (2013). Rotavirus Gastroenteritis in Children under 5 Years in the Kingdom of Bahrain: Hospital-Based Surveillance. Clin. Epidemiol..

[55] Mathew S., Al Ansari K., Al Thani A.A., Zaraket H., Yassine H.M. (2021). Epidemiological, Molecular, and Clinical Features of Rotavirus Infections among Pediatrics in Qatar. Eur. J. Clin. Microbiol. Infect. Dis..

[56] Goodman A. (2015). The Development of the Qatar Healthcare System: A Review of the Literature. Int. J. Clin. Med..

[57] Iaconelli M., Valdazo-González B., Equestre M., Ciccaglione A.R., Marcantonio C., Della Libera S., La Rosa G. (2017). Molecular Characterization of Human Adenoviruses in Urban Wastewaters Using next Generation and Sanger Sequencing. Water Res..

