# Supplementary material for: Molecular Epidemiology and Surveillance of Human Adenovirus and Rotavirus A Associated Gastroenteritis in Riyadh, Saudi Arabia
Source: Trop Med Infect Dis. 2023 May 15;8(5):279. doi: 10.3390/tropicalmed8050279 (PMC10221376; doi:10.3390/tropicalmed8050279)
Supplement: Supplementary file 1 [file tropicalmed-08-00279-s001.zip › TMID-supplementary.pdf]

Tropical Medicine and Infectious Disease

MDPI

Supplementary Materials:

Table S1. Evolutionary divergence estimates between Sequences of HRV

Species 1	Species 2	Distance (*d*)
SAU/SA-01/10.24.17	SAU/SA-02/01.20.18	0.060317
**SAU/SA-01/10.24.17**	IRA/50/2016/G2	0.005807
**SAU/SA-01/10.24.17**	TUR/TOKAT/2016/G2	0.005807
**SAU/SA-01/10.24.17**	TUR/FYON/2015/G2	0.005807
**SAU/SA-01/10.24.17**	CHN/SH-RV76/2015/G2P4	0.005807
**SAU/SA-01/10.24.17**	KOR/Seoul1602/2011/G2P8	0.005807
**SAU/SA-01/10.24.17**	RUS/O1270/2011/G2P8	0.005807
**SAU/SA-01/10.24.17**	RUS/Nov11-N1936/2011/G2P8	0.005807
**SAU/SA-01/10.24.17**	RUS/O806/2011/G2P4	0.005807
**SAU/SA-01/10.24.17**	THA/B4285/2017/G2P8	0.005807
SAU/SA-01/10.24.17	THA/B5686/2018/G2P4	0.011660
SAU/SA-01/10.24.17	THA/B5581/2018/G2P8	0.011660
SAU/SA-01/10.24.17	THA/B4360/2017/G2P4	0.011660
SAU/SA-01/10.24.17	RUS/NS18-A1455/2018/G2P4	0.011660
SAU/SA-01/10.24.17	RUS/NS18-A1454/2018/G2P4	0.011660
**SAU/SA-01/10.24.17**	CHN/G12021182/2012/G2P4	0.005807
**SAU/SA-01/10.24.17**	BEL/BE34/2006/G2P4	0.005807
**SAU/SA-01/10.24.17**	IDN/BL-5210/2006/G2P4	0.005807
**SAU/SA-01/10.24.17**	CHN/G17081040/2017/G2P4	0.005807
SAU/SA-01/10.24.17	PAK350/2015/G2P4	0.011660
SAU/SA-01/10.24.17	ETH/BD526/2016/G2P4	0.017594
SAU/SA-01/10.24.17	BRA/IP-393 MG/2010/G2P4	0.011660
SAU/SA-01/10.24.17	BGN/J263/2010/G2P4	0.011660
SAU/SA-01/10.24.17	BEL/BE97/2012/G2P4	0.011660
SAU/SA-01/10.24.17	URY/A70-TyT-09/2011/G2P4	0.011660
SAU/SA-01/10.24.17	BGD/Bang-082/2007/G2P4	0.011660
SAU/SA-01/10.24.17	IND/HR-353/2014/G2P4	0.011667
SAU/SA-01/10.24.17	IND/KOL-18-10/2010/G2P8	0.011660
SAU/SA-01/10.24.17	COD/KisB546/2009/G2P4	0.011660
SAU/SA-01/10.24.17	CAN/RT008-09/2009/G2P4	0.011660
SAU/SA-01/10.24.17	ARG/Arg2850/2010/G2P4	0.011660
SAU/SA-01/10.24.17	ECU/2012826166/2011/G2P8	0.011660
SAU/SA-01/10.24.17	AUS/CK20035/2006/G2P4	0.011660
SAU/SA-01/10.24.17	THA/B4940/2018/G2P4	0.011660
SAU/SA-01/10.24.17	PAK/NIBGE-45/2010/G2P6	0.011660

Tropical Medicine and Infectious Disease

MDPI

SAU/SA-01/10.24.17	BGD/Dhaka28-06/2006/G2P4	0.011660
SAU/SA-01/10.24.17	LBN/R060/2011/G2P4	0.017594
SAU/SA-01/10.24.17	RUS/Nov10-N85/2010/G2P8	0.017594
SAU/SA-01/10.24.17	VEN/CCS-42/2007/G2P4	0.017594
SAU/SA-01/10.24.17	BGN/J253/2010/G2P4	0.017594
SAU/SA-01/10.24.17	IDN/DSA61/2018/G2	0.023613
SAU/SA-01/10.24.17	THA/B4824/2018 G2P4	0.017594
SAU/SA-01/10.24.17	SAU/Taif-1/2013/G1P8	0.234866
SAU/SA-01/10.24.17	SAU/Taif-5/2013/G1P8	0.252203
SAU/SA-01/10.24.17	LBN/A167/2013/G1P8	0.261275
SAU/SA-01/10.24.17	SA/MD28/2007/G9P8	0.309554
SAU/SA-01/10.24.17	LBN/H256/2013/G9P8	0.309554
SAU/SA-01/10.24.17	USA/CNMC122/2011/G12P8	0.345199
**SAU/SA-02/01.20.18**	IRA/50/2016/G2	0.054032
**SAU/SA-02/01.20.18**	TUR/TOKAT/2016/G2	0.054032
**SAU/SA-02/01.20.18**	TUR/FYON/2015/G2	0.054032
**SAU/SA-02/01.20.18**	CHN/SH-RV76/2015/G2P4	0.054032
**SAU/SA-02/01.20.18**	KOR/Seoul1602/2011/G2P8	0.054032
**SAU/SA-02/01.20.18**	RUS/O1270/2011/G2P8	0.054032
**SAU/SA-02/01.20.18**	RUS/Nov11-N1936/2011/G2P8	0.054032
**SAU/SA-02/01.20.18**	RUS/O806/2011/G2P4	0.054032
**SAU/SA-02/01.20.18**	THA/B4285/2017/G2P8	0.054032
SAU/SA-02/01.20.18	THA/B5686/2018/G2P4	0.060271
SAU/SA-02/01.20.18	THA/B5581/2018/G2P8	0.060271
SAU/SA-02/01.20.18	THA/B4360/2017/G2P4	0.060271
SAU/SA-02/01.20.18	RUS/NS18-A1455/2018/G2P4	0.060271
SAU/SA-02/01.20.18	RUS/NS18-A1454/2018/G2P4	0.060271
**SAU/SA-02/01.20.18**	CHN/G12021182/2012/G2P4	0.054032
**SAU/SA-02/01.20.18**	BEL/BE34/2006/G2P4	0.054032
**SAU/SA-02/01.20.18**	IDN/BL-5210/2006/G2P4	0.054032
**SAU/SA-02/01.20.18**	CHN/G17081040/2017/G2P4	0.054032
SAU/SA-02/01.20.18	PAK350/2015/G2P4	0.060271
SAU/SA-02/01.20.18	ETH/BD526/2016/G2P4	0.060275
SAU/SA-02/01.20.18	BRA/IP-393 MG/2010/G2P4	0.060279
SAU/SA-02/01.20.18	BGN/J263/2010/G2P4	0.060271
SAU/SA-02/01.20.18	BEL/BE97/2012/G2P4	0.060279
SAU/SA-02/01.20.18	URY/A70-TyT-09/2011/G2P4	0.060279
SAU/SA-02/01.20.18	BGD/Bang-082/2007/G2P4	0.060271
SAU/SA-02/01.20.18	IND/HR-353/2014/G2P4	0.060310
SAU/SA-02/01.20.18	IND/KOL-18-10/2010/G2P8	0.060271

SAU/SA-02/01.20.18	COD/KisB546/2009/G2P4	0.060271
SAU/SA-02/01.20.18	CAN/RT008-09/2009/G2P4	0.060279
SAU/SA-02/01.20.18	ARG/Arg2850/2010/G2P4	0.060279
SAU/SA-02/01.20.18	ECU/2012826166/2011/G2P8	0.060271
SAU/SA-02/01.20.18	AUS/CK20035/2006/G2P4	0.060279
SAU/SA-02/01.20.18	THA/B4940/2018/G2P4	0.060271
SAU/SA-02/01.20.18	PAK/NIBGE-45/2010/G2P6	0.060271
SAU/SA-02/01.20.18	BGD/Dhaka28-06/2006/G2P4	0.060271
SAU/SA-02/01.20.18	LBN/R060/2011/G2P4	0.066610
SAU/SA-02/01.20.18	RUS/Nov10-N85/2010/G2P8	0.066610
SAU/SA-02/01.20.18	VEN/CCS-42/2007/G2P4	0.066610
SAU/SA-02/01.20.18	BGN/J253/2010/G2P4	0.066610
SAU/SA-02/01.20.18	IDN/DSA61/2018/G2	0.066616
SAU/SA-02/01.20.18	THA/B4824/2018 G2P4	0.066610
SAU/SA-02/01.20.18	SAU/Taif-1/2013/G1P8	0.260027
SAU/SA-02/01.20.18	SAU/Taif-5/2013/G1P8	0.278009
SAU/SA-02/01.20.18	LBN/A167/2013/G1P8	0.287460
SAU/SA-02/01.20.18	SA/MD28/2007/G9P8	0.313324
SAU/SA-02/01.20.18	LBN/H256/2013/G9P8	0.313324
SAU/SA-02/01.20.18	USA/CNMC122/2011/G12P8_{outgroup}	0.330552

The count of base substitutions per site from inter- sequences are displayed. Final dataset included a total of 173 positions. The numbers in red referred to the closest distance (least evolutionary divergence for each sequence. The first bracket denoted the closest sequences to SAU/SA-01/10.24.17 (bold), whereas the second bracket refers to sequences closer to SAU/SA-02/01.20.18 (bold).

Tropical Medicine and Infectious Disease

Table S2. Sequences used for phylogenetic analysis of HRV

Accession number	Sequence nomination	Abbreviated name
MK050144	Rotavirus A isolate Human/50/Iran/2016/G2 VP7 (VP7) gene	IRA/50/2016/G2
MF494809	Rotavirus G2 isolate 93TOKAT2016/G2 capsid glycoprotein VP7 (VP7) gene	TUR/TOKAT/2016/G2
MF494793	Rotavirus G2 isolate 13AFYON2015/G2 capsid glycoprotein VP7 (VP7) gene	TUR/FYON/2015/G2
KU532216	Human rotavirus A isolate RVA/Human-wt/CHN/SH-RV76/2015/G2P[4] major outer capsid protein VP7 gene	CHN/SH-RV76/2015/G2P4
KF812584	Human rotavirus A isolate Human/KOR/Seoul1602/2011/G2P[8] VP7 (VP7) gene	KOR/SEOUL1602/2011/G2P8
KF006866	Human rotavirus A isolate Hu/RUS/O1270/2011/G2P[8] outer capsid protein VP7 (VP7) gene	RUS/O1270/2011/G2P8
JX841121	Rotavirus A isolate Hu/RUS/Nov11-N1936/2011/G2P[8] outer capsid protein VP7 (VP7) gene	RUS/Nov11-N1936/2011/G2P8
JX156394	Human rotavirus A isolate Hu/RUS/O806/2011/G2P[4] outer capsid protein VP7 (VP7) gene	RUS/O806/2011/G2P4
MN836878	Human rotavirus isolate THA/B4285/2017_G2P[8] outer capsid glycoprotein VP7 gene	THA/B4285/2017/G2P8
MW058340	Human rotavirus isolate THA/B5686/2018_G2P[4] outer capsid glycoprotein VP7 gene	THA/B5686/2018/G2P4
MW058329	Human rotavirus isolate THA/B5581/2018_G2P[8] outer capsid glycoprotein VP7 gene	THA/B5581/2018/G2P4
MW058176	Human rotavirus isolate THA/B4360/2017_G2P[4] outer capsid glycoprotein VP7 gene	THA/B4360/2018/G2P4
MN577199	Rotavirus A isolate RVA/Human-wt/RUS/Novosibirsk/NS18-A1455/2018/G2P[4] outer capsid protein (VP7) gene	RUS/NS18-A1455/2018/G2P4
MN577198	Rotavirus A isolate RVA/Human-wt/RUS/Novosibirsk/NS18-A1454/2018/G2P[4] outer capsid protein (VP7) gene	RUS/NS18-A1454/2018/G2P4
MH625736	Rotavirus A strain RVA/Human-wt/CHN/G12021182/2012/G2P[4] outer capsid protein VP7 gene	CHN/G12021182/2012/G2P4
KR705272	Rotavirus A strain RVA/Human-wt/BEL/BE34/2006/G2P[4] segment 9 VP7 (VP7) gene	BEL/BE34/2006/G2P6
JQ837882	Rotavirus A strain RVA/Human-wt/IDN/BL-5210/2006/G2P[4] outer capsid protein VP7 (VP7) gene	IDN/BL-5210/2006/G2P4
MN856459	Rotavirus A isolate RVA/Human-wt/CHN/G17081040/2017/G2P[4] outer capsid protein VP7 (VP7) gene	CHN/G17081040/2017/G2P4
MH182475	Human rotavirus A strain RVA/Human-wt/PAK350/2015/G2P4 segment 9 outer capsid glycoprotein VP7 (VP7) gene	PAK/PAK350/2015/G2P4
MH382858	Rotavirus A strain RVA/Human-wt/ETH/BD526/2016/G2P[4] outer capsid glycoprotein VP7 gene	ETH/BD526/2016/G2P4
KF938703	Human rotavirus A strain RVA/Human-wt/BRA/IP-393_MG/2010/G2P[4] outer capsid protein (VP7) gene	BRA/IP-393-MG/2010/G2P4
KU248421	Human rotavirus A strain RVA/Human-wt/BGN/J263/2010/G2P[4] VP7 gene	BGN/J263/2010/G2P4
KR705437	Rotavirus A strain RVA/Human-wt/BEL/BE97/2012/G2P[4] segment 9 VP7 (VP7) gene	BEL/BE97/2012/G2P4
KP271247	Rotavirus A strain RVA/Env/URY/A70-TyT-09/2011/G2P4 VP7 (VP7) gene	URY/A70-TyT-09/2011/G2P4

KP882076	Rotavirus A strain RVA/Human-wt/BGD/Bang-082/2007/G2P[4] segment 9 VP7 (VP7) gene	BGD/Bang-082/2007/G2P4
KP054260	Rotavirus A isolate HRVA/HR-353/G2P[4]/CMBT/MDU/Rohtak VP7 (VP7) gene	IND/HR-353/2014/G2P4
KM008646	Rotavirus A strain RVA/Human-wt/IND/KOL-18-10/2010/G2P[8] glycoprotein gene	IND/KOL-18-10/2010/G2P8
KJ870843	Rotavirus A strain RVA/Human-wt/COD/KisB546/2009/G2P[4] VP7 gene	COD/KisB546/2009/G2P4
JQ069521	Human rotavirus A strain RVA/Human-wt/CAN/RT008-09/2009/G2P4 outer capsid surface protein (G) gene	CAN/RT008-09/2009/G2P4
KF920558	Rotavirus A RVA/Human-wt/ARG/Arg2850/2010/G2P[4] VP7 gene	ARG/Arg2850/2010/G2P4
KC951932	Rotavirus A isolate RVA/Human-wt/ECU/2012826166/2011/G2P[8] VP7 (VP7) gene	ECU/2012826166/2011/G2P8
KC443405	Rotavirus A strain RVA/Human-wt/AUS/CK20035/2006/G2P[4] segment 9 capsid glycoprotein VP7 (VP7) gene	AUS/CK20035/2006/G2P4
MN836952	Human rotavirus isolate THA/B4940/2018_G2P[4] outer capsid glycoprotein VP7 gene	THA/B4940/2018/G2P4
JN001883	Rotavirus A strain human-wt/PAK/NIBGE-45/2010/G2P6 outer capsid protein VP7 gene	PAK/NIBGE-45/2010/G2P6
EF690791	Rotavirus A strain Hu/G2P[4]/Dhaka28-06/2006/BGD VP7 (VP7) gene	BGD/Dhaka28-06/2006/G2P4
MH591273	Rotavirus A strain RVA/Human-wt/LBNR060/2011/G2P[4] glycoprotein (VP7) gene	LBN/R060/2011/G2P4
MG932619	Human rotavirus A strain RVA/Hu-wt/RUS/Novosibirsk/Nov10-N85/2010/G2P[8] outer capsid protein VP7 (VP7) gene	RUS/Nov10-N85/2010/G2P8
KY053848	Human rotavirus A strain RVA/Human-wt/VEN/CCS-42/2007/G2P[4] glycoprotein (VP7) gene	VEN/CCS-42/2007/G2P4
KU356579	Human rotavirus A strain RVA/Human-wt/BGN/J253/2010/G2P[4] VP7 gene	BGN/J253/2010/G2P4
LC597290	Rotavirus A RVA/Human-wt/IDN/DSA61/2018/G2 gene for outer capsid glycoprotein	IDN/DSA61/2018/G2
MN836934	Human rotavirus isolate THA/B4824/2018_G2P[4] outer capsid glycoprotein VP7 gene	THA/B4824/2018/G2P4
KP281273	Human rotavirus A isolate RVA/Human/SAU/Taif-1/2013/G1P[8] VP7	SAU/Taif-1/2013/G1P8
KP281277	Human rotavirus A isolate RVA/Human/SAU/Taif-5/2013/G1P[8] VP7	SAU/Taif-5/2013/G1P8
MH591261	Rotavirus A strain RVA/Human-wt/LBNA167/2013/G1P[8] glycoprotein (VP7) gene	LBN/A167/2013/G1P8
MH591313	Rotavirus A strain RVA/Human-wt/LBNH256/2013/G9P[8] glycoprotein (VP7) gene	LBN/H256/2013/G9P8
AB297791	Human rotavirus A gene for VP7, strain: MD28	SA/MD28/2007/G9P8
KT920656	Rotavirus A strain RVA/Human-wt/USA/CNMC122/2011/G12P[8] segment 9 capsid glycoprotein VP7 (VP7) gene	USA/CNMC122/2011/G12P8 {Outgroup}

Table S3. Best fitting model selection for HRV using Maximum Likelihood fits of 24 different nucleotide substitution models

Model	#Param	BIC	AICc	lnL	Invariant	Gamma	R	Freq A	Freq T	Freq C	Freq G	A=>T	A=>C	A=>G	T=>A	T=>C	T=>G	C=>A	C=>T	C=>G	G=>A	G=>T	G=>C
**T92+I***	98	**2351.5**	1663.5	-732.6	0.517	n/a	3.030	0.342	0.342	0.158	0.158	0.04	0.02	0.12	0.04	0.12	0.02	0.04	0.27	0.02	0.27	0.04	0.02
T92+G+I	99	2360.6	1665.6	-732.6	0.515	200	3.037	0.342	0.342	0.158	0.158	0.04	0.02	0.12	0.04	0.12	0.02	0.04	0.27	0.02	0.27	0.04	0.02
T92+G	98	2363.7	1675.6	-738.7	n/a	0.802	2.695	0.342	0.342	0.158	0.158	0.04	0.02	0.12	0.04	0.12	0.02	0.04	0.26	0.02	0.26	0.04	0.02
HKY+I	100	2365.7	1663.7	-730.6	0.517	n/a	3.044	0.363	0.321	0.133	0.182	0.04	0.01	0.14	0.04	0.1	0.02	0.04	0.25	0.02	0.28	0.04	0.01
TN93+I	101	2374.5	1665.5	-730.5	0.516	n/a	3.060	0.363	0.321	0.133	0.182	0.04	0.01	0.15	0.04	0.09	0.02	0.04	0.23	0.02	0.3	0.04	0.01
HKY+G+I	101	2374.8	1665.7	-730.6	0.516	200	3.048	0.363	0.321	0.133	0.182	0.04	0.01	0.14	0.04	0.1	0.02	0.04	0.25	0.02	0.28	0.04	0.01
HKY+G	100	2378.1	1676.1	-736.9	n/a	0.802	2.676	0.363	0.321	0.133	0.182	0.04	0.02	0.14	0.04	0.1	0.02	0.04	0.24	0.02	0.27	0.04	0.02
TN93+G+I	102	2383.7	1667.7	-730.6	0.514	200	3.128	0.363	0.321	0.133	0.182	0.04	0.01	0.16	0.04	0.09	0.02	0.04	0.22	0.02	0.31	0.04	0.01
T92	97	2386.2	1705.2	-754.5	n/a	n/a	2.162	0.342	0.342	0.158	0.158	0.05	0.02	0.11	0.05	0.11	0.02	0.05	0.24	0.02	0.24	0.05	0.02
TN93+G	101	2387.0	1678.0	-736.8	n/a	0.802	2.684	0.363	0.321	0.133	0.182	0.04	0.02	0.14	0.05	0.09	0.02	0.05	0.23	0.02	0.29	0.04	0.02
K2+I	97	2389.7	1708.7	-756.2	0.512	n/a	2.502	0.25	0.25	0.25	0.25	0.04	0.04	0.18	0.04	0.18	0.04	0.04	0.18	0.04	0.18	0.04	0.04
GTR+I	104	2398.0	1668.0	-728.7	0.515	n/a	3.022	0.363	0.321	0.133	0.182	0.03	0.03	0.15	0.03	0.09	0.01	0.09	0.23	0.01	0.29	0.03	0.01
K2+G+I	98	2398.8	1710.8	-756.2	0.511	200	2.507	0.25	0.25	0.25	0.25	0.04	0.04	0.18	0.04	0.18	0.04	0.04	0.18	0.04	0.18	0.04	0.04
K2+G	97	2398.9	1717.8	-760.8	n/a	0.802	2.375	0.25	0.25	0.25	0.25	0.04	0.04	0.18	0.04	0.18	0.04	0.04	0.18	0.04	0.18	0.04	0.04
HKY	99	2400.5	1705.4	-752.5	n/a	n/a	2.150	0.363	0.321	0.133	0.182	0.05	0.02	0.13	0.05	0.09	0.03	0.05	0.23	0.03	0.26	0.05	0.02
GTR+G+I	105	2407.0	1670.1	-728.7	0.514	200	3.027	0.363	0.321	0.133	0.182	0.03	0.03	0.15	0.03	0.09	0.01	0.09	0.23	0.01	0.29	0.03	0.01
TN93	100	2409.2	1707.1	-752.4	n/a	n/a	2.147	0.363	0.321	0.133	0.182	0.05	0.02	0.13	0.05	0.09	0.03	0.05	0.22	0.03	0.27	0.05	0.02
GTR+G	104	2410.2	1680.2	-734.8	n/a	0.802	2.633	0.363	0.321	0.133	0.182	0.03	0.03	0.14	0.04	0.1	0.01	0.09	0.23	0.02	0.28	0.02	0.01
K2	96	2416.7	1742.7	-774.2	n/a	n/a	2.053	0.25	0.25	0.25	0.25	0.04	0.04	0.17	0.04	0.17	0.04	0.04	0.17	0.04	0.17	0.04	0.04
GTR	103	2432.6	1709.6	-750.5	n/a	n/a	2.134	0.363	0.321	0.133	0.182	0.05	0.03	0.13	0.05	0.09	0.02	0.09	0.22	0.02	0.26	0.03	0.01
JC+I	96	2435.0	1761.0	-783.4	0.493	n/a	0.5	0.25	0.25	0.25	0.25	0.08	0.08	0.08	0.08	0.08	0.08	0.08	0.08	0.08	0.08	0.08	0.08
JC+G	96	2442.9	1768.8	-787.3	n/a	0.802	0.5	0.25	0.25	0.25	0.25	0.08	0.08	0.08	0.08	0.08	0.08	0.08	0.08	0.08	0.08	0.08	0.08
JC+G+I	97	2444.1	1763.1	-783.4	0.493	200	0.5	0.25	0.25	0.25	0.25	0.08	0.08	0.08	0.08	0.08	0.08	0.08	0.08	0.08	0.08	0.08	0.08
JC	95	2456.2	1789.2	-798.5	n/a	n/a	0.5	0.25	0.25	0.25	0.25	0.08	0.08	0.08	0.08	0.08	0.08	0.08	0.08	0.08	0.08	0.08	0.08

*Models with the lowest BIC scores (Bayesian Information Criterion) are considered to describe the substitution pattern the best (written in red and bold). For each model, AICc value (Akaike Information Criterion, corrected), Maximum Likelihood value (lnL), and the number of parameters (including

branch lengths) are also depicted above. 5 rate categories-discrete Gamma distribution (+*G*) was applied for modeling the evolutionary rates non-uniformity among sites and supposing that a certain fraction of sites is evolutionarily invariable (+*I*). Proposed or estimated values of transition/transversion bias (R) for each model are displayed. They are followed by nucleotide frequencies (*f*) and rates of base substitutions (*r*) for each nucleotide pair.

* Abbreviations: TR: General Time Reversible; HKY: Hasegawa-Kishino-Yano; TN93: Tamura-Nei; T92: Tamura 3-parameter; K2: Kimura 2-parameter; JC: Jukes-Cantor.

Tropical Medicine and Infectious Disease

MDPI

Table S4. Evolutionary divergence estimates between Sequences of HAdV

Species 1	Species 2	Distance (*d*)
HAdV/SAU/1.23.18	HAdV/BRA /IAL-AD89/2014/41	0.027311
HAdV/SAU/1.23.18	HAdV/CHN/FX1-152772/2015/41	0.035301
HAdV/SAU/1.23.18	HAdV/SAU/SA12680/2014/41	0.035301
HAdV/SAU/1.23.18	HAdV/SWD/GyK253/2000/41	0.035301
HAdV/SAU/1.23.18	HAdV/CHN/D363/2015/41	0.035301
HAdV/SAU/1.23.18	HAdV/USA/4849/2010/41	0.035301
HAdV/SAU/1.23.18	HAdV/TUR/ANKARA10/2016/41	0.035301
HAdV/SAU/1.23.18	HAdV/FRA/MU22/2018/41	0.039327
HAdV/SAU/1.23.18	HAdV/NETH/Tak/2007/41	0.039327
HAdV/SAU/1.23.18	HAdV/BRA/IAL-AD694/2016/40	0.072932
HAdV/SAU/1.23.18	HAdV/CHN/GZ20191121/026/2019/E4_{outgroup}	0.161870
HAdV/SAU/1.23.18	HAdV/BRA/IAL-AD178/2016/41	0.031295
HAdV/SAU/1.23.18	HAdV/IRQ/MU35/2016/41	0.039327
HAdV/SAU/1.23.18	HAdV/IND/BDF/2017/41	0.035301
HAdV/SAU/1.23.18	HAdV/CHN/D240/2015/41	0.035301
HAdV/SAU/1.23.18	HAdV/JPN/SaP3-3F/2012/41	0.035301
HAdV/SAU/1.23.18	HAdV/IND/503214/2009/41	0.035301
HAdV/SAU/1.23.18	HAdv/FIN/HoviX/1979/40	0.081160
HAdV/SAU/1.23.18	HAdV/BRA/IAL-AD09/2016/41	0.031295
HAdV/SAU/1.23.18	HAdV/IND/SWR9/2017/41	0.035301
HAdV/SAU/1.23.18	HAdV/JPN/D27/2011/41	0.035301
HAdV/SAU/1.23.18	HAdV/CHN/NIVD103/2007/41	0.035301
HAdV/SAU/1.23.18	HAdV/SAU/SA12303/2014/40	0.081160
HAdV/SAU/1.23.18	HAdV/JPN/Dugan/2007/40	0.081160
HAdV/SAU/1.23.18	HAdV/CHN/D240/2015/41	0.035301
HAdV/SAU/1.23.18	HAdV/CHN/D4/2011/41	0.035301
HAdV/SAU/1.23.18	HAdV/UK/Dugan/1990/40	0.081160
HAdV/SAU/1.23.18	HAdV/SAU/SA6749/2014/41	0.043376
HAdV/SAU/1.23.18	HAdV/BRA/IAL-AD99/2015/40	0.072345
HAdV/SAU/1.23.18	HAdV/USA/4845/2010/41	0.035301
HAdV/SAU/1.23.18	HAdV/USA/HS12/2009/41	0.035301
**HAdV/SAU/10.12.17**	HAdV/SAU/10.25.17	0.003841
HAdV/SAU/10.12.17	HAdV/SAU/11.25.17	0.015484
HAdV/SAU/10.12.17	HAdV/SAU/12.13.17	0.011583
HAdV/SAU/10.12.17	HAdV/SAU/1.23.18	0.027311
HAdV/SAU/10.12.17	HAdV/BRA /IAL-AD89/2014/41	0.007702
HAdV/SAU/10.12.17	HAdV/CHN/FX1-152772/2015/41	0.015484
HAdV/SAU/10.12.17	HAdV/SAU/SA12680/2014/41	0.015484

Tropical Medicine and Infectious Disease

MDPI

HAdV/SAU/10.12.17	HAdV/SWD/GyK253/2000/41	0.015484
HAdV/SAU/10.12.17	HAdV/CHN/D363/2015/41	0.015484
HAdV/SAU/10.12.17	HAdV/USA/4849/2010/41	0.015484
HAdV/SAU/10.12.17	HAdV/TUR/ANKARA10/2016/41	0.015484
HAdV/SAU/10.12.17	HAdV/FRA/MU22/2018/41	0.019406
HAdV/SAU/10.12.17	HAdV/NETH/Tak/2007/41	0.019406
HAdV/SAU/10.12.17	HAdV/BRA/IAL-AD694/2016/40	0.043721
HAdV/SAU/10.12.17	HAdV/CHN/GZ20191121/026/2019/E4_{outgroup}	0.133873
**HAdV/SAU/10.12.17**	HAdV/BRA/IAL-AD178/2016/41	0.003841
HAdV/SAU/10.12.17	HAdV/IRQ/MU35/2016/41	0.019406
HAdV/SAU/10.12.17	HAdV/IND/BDF/2017/41	0.015484
HAdV/SAU/10.12.17	HAdV/CHN/D240/2015/41	0.015484
HAdV/SAU/10.12.17	HAdV/JPN/SaP3-3F/2012/41	0.015484
HAdV/SAU/10.12.17	HAdV/IND/503214/2009/41	0.015484
HAdV/SAU/10.12.17	HAdv/FIN/HoviX/1979/40	0.051745
HAdV/SAU/10.12.17	HAdV/BRA/IAL-AD09/2016/41	0.007702
HAdV/SAU/10.12.17	HAdV/IND/SWR9/2017/41	0.015484
HAdV/SAU/10.12.17	HAdV/JPN/D27/2011/41	0.015484
HAdV/SAU/10.12.17	HAdV/CHN/NIVD103/2007/41	0.015484
HAdV/SAU/10.12.17	HAdV/SAU/SA12303/2014/40	0.051745
HAdV/SAU/10.12.17	HAdV/JPN/Dugan/2007/40	0.051745
HAdV/SAU/10.12.17	HAdV/CHN/D240/2015/41	0.015484
HAdV/SAU/10.12.17	HAdV/CHN/D4/2011/41	0.015484
HAdV/SAU/10.12.17	HAdV/UK/Dugan/1990/40	0.051745
HAdV/SAU/10.12.17	HAdV/SAU/SA6749/2014/41	0.023348
HAdV/SAU/10.12.17	HAdV/BRA/IAL-AD99/2015/40	0.043376
HAdV/SAU/10.12.17	HAdV/USA/4845/2010/41	0.015484
HAdV/SAU/10.12.17	HAdV/USA/HS12/2009/41	0.015484
HAdV/SAU/10.25.17	HAdV/SAU/11.25.17	0.011583
HAdV/SAU/10.25.17	HAdV/SAU/12.13.17	0.007702
HAdV/SAU/10.25.17	HAdV/SAU/1.23.18	0.031295
**HAdV/SAU/10.25.17**	HAdV/BRA /IAL-AD89/2014/41	0.003841
HAdV/SAU/10.25.17	HAdV/CHN/FX1-152772/2015/41	0.011583
HAdV/SAU/10.25.17	HAdV/SAU/SA12680/2014/41	0.011583
HAdV/SAU/10.25.17	HAdV/SWD/GyK253/2000/41	0.011583
HAdV/SAU/10.25.17	HAdV/CHN/D363/2015/41	0.011583
HAdV/SAU/10.25.17	HAdV/USA/4849/2010/41	0.011583
HAdV/SAU/10.25.17	HAdV/TUR/ANKARA10/2016/41	0.011583
HAdV/SAU/10.25.17	HAdV/FRA/MU22/2018/41	0.015484
HAdV/SAU/10.25.17	HAdV/NETH/Tak/2007/41	0.015484
HAdV/SAU/10.25.17	HAdV/BRA/IAL-AD694/2016/40	0.039639
HAdV/SAU/10.25.17	HAdV/CHN/GZ20191121/026/2019/E4_{outgroup}	0.129307
**HAdV/SAU/10.25.17**	**HAdV/BRA/IAL-AD178/2016/41**	0.000000
HAdV/SAU/10.25.17	HAdV/IRQ/MU35/2016/41	0.015484
HAdV/SAU/10.25.17	HAdV/IND/BDF/2017/41	0.011583

HAdV/SAU/10.25.17	HAdV/CHN/D240/2015/41	0.011583
HAdV/SAU/10.25.17	HAdV/JPN/SaP3-3F/2012/41	0.011583
HAdV/SAU/10.25.17	HAdV/IND/503214/2009/41	0.011583
HAdV/SAU/10.25.17	HAdv/FIN/HoviX/1979/40	0.047635
**HAdV/SAU/10.25.17**	HAdV/BRA/IAL-AD09/2016/41	0.003841
HAdV/SAU/10.25.17	HAdV/IND/SWR9/2017/41	0.011583
HAdV/SAU/10.25.17	HAdV/JPN/D27/2011/41	0.011583
HAdV/SAU/10.25.17	HAdV/CHN/NIVD103/2007/41	0.011583
HAdV/SAU/10.25.17	HAdV/SAU/SA12303/2014/40	0.047635
HAdV/SAU/10.25.17	HAdV/JPN/Dugan/2007/40	0.047635
HAdV/SAU/10.25.17	HAdV/CHN/D240/2015/41	0.011583
HAdV/SAU/10.25.17	HAdV/CHN/D4/2011/41	0.011583
HAdV/SAU/10.25.17	HAdV/UK/Dugan/1990/40	0.047635
HAdV/SAU/10.25.17	HAdV/SAU/SA6749/2014/41	0.019406
HAdV/SAU/10.25.17	HAdV/BRA/IAL-AD99/2015/40	0.039327
HAdV/SAU/10.25.17	HAdV/USA/4845/2010/41	0.011583
HAdV/SAU/10.25.17	HAdV/USA/HS12/2009/41	0.011583
HAdV/SAU/11.25.17	HAdV/SAU/12.13.17	0.019406
HAdV/SAU/11.25.17	HAdV/SAU/1.23.18	0.043376
HAdV/SAU/11.25.17	HAdV/BRA /IAL-AD89/2014/41	0.015484
HAdV/SAU/11.25.17	HAdV/CHN/FX1-152772/2015/41	0.023348
HAdV/SAU/11.25.17	HAdV/SAU/SA12680/2014/41	0.023348
HAdV/SAU/11.25.17	HAdV/SWD/GyK253/2000/41	0.023348
HAdV/SAU/11.25.17	HAdV/CHN/D363/2015/41	0.023348
HAdV/SAU/11.25.17	HAdV/USA/4849/2010/41	0.023348
HAdV/SAU/11.25.17	HAdV/TUR/ANKARA10/2016/41	0.023348
HAdV/SAU/11.25.17	HAdV/FRA/MU22/2018/41	0.027311
HAdV/SAU/11.25.17	HAdV/NETH/Tak/2007/41	0.027311
HAdV/SAU/11.25.17	HAdV/BRA/IAL-AD694/2016/40	0.051952
HAdV/SAU/11.25.17	HAdV/CHN/GZ20191121/026/2019/E4_{outgroup}	0.143090
HAdV/SAU/11.25.17	HAdV/BRA/IAL-AD178/2016/41	0.011583
HAdV/SAU/11.25.17	HAdV/IRQ/MU35/2016/41	0.027311
HAdV/SAU/11.25.17	HAdV/IND/BDF/2017/41	0.023348
HAdV/SAU/11.25.17	HAdV/CHN/D240/2015/41	0.023348
HAdV/SAU/11.25.17	HAdV/JPN/SaP3-3F/2012/41	0.023348
HAdV/SAU/11.25.17	HAdV/IND/503214/2009/41	0.023348
HAdV/SAU/11.25.17	HAdv/FIN/HoviX/1979/40	0.060032
HAdV/SAU/11.25.17	HAdV/BRA/IAL-AD09/2016/41	0.015484
HAdV/SAU/11.25.17	HAdV/IND/SWR9/2017/41	0.023348
HAdV/SAU/11.25.17	HAdV/JPN/D27/2011/41	0.023348
HAdV/SAU/11.25.17	HAdV/CHN/NIVD103/2007/41	0.023348
HAdV/SAU/11.25.17	HAdV/SAU/SA12303/2014/40	0.060032
HAdV/SAU/11.25.17	HAdV/JPN/Dugan/2007/40	0.060032
HAdV/SAU/11.25.17	HAdV/CHN/D240/2015/41	0.023348
HAdV/SAU/11.25.17	HAdV/CHN/D4/2011/41	0.023348

HAdV/SAU/11.25.17	HAdV/UK/Dugan/1990/40	0.060032
HAdV/SAU/11.25.17	HAdV/SAU/SA6749/2014/41	0.031295
HAdV/SAU/11.25.17	HAdV/BRA/IAL-AD99/2015/40	0.051539
HAdV/SAU/11.25.17	HAdV/USA/4845/2010/41	0.023348
HAdV/SAU/11.25.17	HAdV/USA/HS12/2009/41	0.023348
HAdV/SAU/12.13.17	HAdV/SAU/1.23.18	0.031295
HAdV/SAU/12.13.17	HAdV/BRA /IAL-AD89/2014/41	0.011583
HAdV/SAU/12.13.17	HAdV/CHN/FX1-152772/2015/41	0.019406
HAdV/SAU/12.13.17	HAdV/SAU/SA12680/2014/41	0.019406
HAdV/SAU/12.13.17	HAdV/SWD/GyK253/2000/41	0.019406
HAdV/SAU/12.13.17	HAdV/CHN/D363/2015/41	0.019406
HAdV/SAU/12.13.17	HAdV/USA/4849/2010/41	0.019406
HAdV/SAU/12.13.17	HAdV/TUR/ANKARA10/2016/41	0.019406
HAdV/SAU/12.13.17	HAdV/FRA/MU22/2018/41	0.023348
HAdV/SAU/12.13.17	HAdV/NETH/Tak/2007/41	0.023348
HAdV/SAU/12.13.17	HAdV/BRA/IAL-AD694/2016/40	0.047825
HAdV/SAU/12.13.17	HAdV/CHN/GZ20191121/026/2019/E4_{outgroup}	0.138467
HAdV/SAU/12.13.17	HAdV/BRA/IAL-AD178/2016/41	0.007702
HAdV/SAU/12.13.17	HAdV/IRQ/MU35/2016/41	0.023348
HAdV/SAU/12.13.17	HAdV/IND/BDF/2017/41	0.019406
HAdV/SAU/12.13.17	HAdV/CHN/D240/2015/41	0.019406
HAdV/SAU/12.13.17	HAdV/JPN/SaP3-3F/2012/41	0.019406
HAdV/SAU/12.13.17	HAdV/IND/503214/2009/41	0.019406
HAdV/SAU/12.13.17	HAdv/FIN/HoviX/1979/40	0.055877
HAdV/SAU/12.13.17	HAdV/BRA/IAL-AD09/2016/41	0.011583
HAdV/SAU/12.13.17	HAdV/IND/SWR9/2017/41	0.019406
HAdV/SAU/12.13.17	HAdV/JPN/D27/2011/41	0.019406
HAdV/SAU/12.13.17	HAdV/CHN/NIVD103/2007/41	0.019406
HAdV/SAU/12.13.17	HAdV/SAU/SA12303/2014/40	0.055877
HAdV/SAU/12.13.17	HAdV/JPN/Dugan/2007/40	0.055877
HAdV/SAU/12.13.17	HAdV/CHN/D240/2015/41	0.019406
HAdV/SAU/12.13.17	HAdV/CHN/D4/2011/41	0.019406
HAdV/SAU/12.13.17	HAdV/UK/Dugan/1990/40	0.055877
HAdV/SAU/12.13.17	HAdV/SAU/SA6749/2014/41	0.027311
HAdV/SAU/12.13.17	HAdV/BRA/IAL-AD99/2015/40	0.047447
HAdV/SAU/12.13.17	HAdV/USA/4845/2010/41	0.019406
HAdV/SAU/12.13.17	HAdV/USA/HS12/2009/41	0.019406
HAdV/SAU/3.31.17	HAdV/SAU/9.15.17	0.035301
HAdV/SAU/3.31.17	HAdV/SAU/10.12.17	0.019406
HAdV/SAU/3.31.17	HAdV/SAU/10.25.17	0.015484
HAdV/SAU/3.31.17	HAdV/SAU/11.25.17	0.027311
HAdV/SAU/3.31.17	HAdV/SAU/12.13.17	0.023348
HAdV/SAU/3.31.17	HAdV/SAU/1.23.18	0.039327
HAdV/SAU/3.31.17	HAdV/BRA /IAL-AD89/2014/41	0.011583
HAdV/SAU/3.31.17	HAdV/CHN/FX1-152772/2015/41	0.019406

HAdV/SAU/3.31.17	HAdV/SAU/SA12680/2014/41	0.019406
HAdV/SAU/3.31.17	HAdV/SWD/GyK253/2000/41	0.019406
HAdV/SAU/3.31.17	HAdV/CHN/D363/2015/41	0.019406
HAdV/SAU/3.31.17	HAdV/USA/4849/2010/41	0.019406
HAdV/SAU/3.31.17	HAdV/TUR/ANKARA10/2016/41	0.019406
HAdV/SAU/3.31.17	HAdV/FRA/MU22/2018/41	0.023348
HAdV/SAU/3.31.17	HAdV/NETH/Tak/2007/41	0.023348
HAdV/SAU/3.31.17	HAdV/BRA/IAL-AD694/2016/40	0.056101
HAdV/SAU/3.31.17	HAdV/CHN/GZ20191121/026/2019/E4_{outgroup}	0.147741
HAdV/SAU/3.31.17	HAdV/BRA/IAL-AD178/2016/41	0.015484
HAdV/SAU/3.31.17	HAdV/IRQ/MU35/2016/41	0.023348
HAdV/SAU/3.31.17	HAdV/IND/BDF/2017/41	0.019406
HAdV/SAU/3.31.17	HAdV/CHN/D240/2015/41	0.019406
HAdV/SAU/3.31.17	HAdV/JPN/SaP3-3F/2012/41	0.019406
HAdV/SAU/3.31.17	HAdV/IND/503214/2009/41	0.019406
HAdV/SAU/3.31.17	HAdV/FIN/HoviX/1979/40	0.064210
HAdV/SAU/3.31.17	HAdV/BRA/IAL-AD09/2016/41	0.015484
HAdV/SAU/3.31.17	HAdV/IND/SWR9/2017/41	0.019406
HAdV/SAU/3.31.17	HAdV/JPN/D27/2011/41	0.019406
HAdV/SAU/3.31.17	HAdV/CHN/NIVD103/2007/41	0.019406
HAdV/SAU/3.31.17	HAdV/SAU/SA12303/2014/40	0.064210
HAdV/SAU/3.31.17	HAdV/JPN/Dugan/2007/40	0.064210
HAdV/SAU/3.31.17	HAdV/CHN/D240/2015/41	0.019406
HAdV/SAU/3.31.17	HAdV/CHN/D4/2011/41	0.019406
HAdV/SAU/3.31.17	HAdV/UK/Dugan/1990/40	0.064210
HAdV/SAU/3.31.17	HAdV/SAU/SA6749/2014/41	0.027311
HAdV/SAU/3.31.17	HAdV/BRA/IAL-AD99/2015/40	0.055655
HAdV/SAU/3.31.17	HAdV/USA/4845/2010/41	0.019406
HAdV/SAU/3.31.17	HAdV/USA/HS12/2009/41	0.019406
HAdV/SAU/9.15.17	HAdV/SAU/10.12.17	0.023348
HAdV/SAU/9.15.17	HAdV/SAU/10.25.17	0.019406
HAdV/SAU/9.15.17	HAdV/SAU/11.25.17	0.031295
HAdV/SAU/9.15.17	HAdV/SAU/12.13.17	0.023348
HAdV/SAU/9.15.17	HAdV/SAU/1.23.18	0.043376
HAdV/SAU/9.15.17	HAdV/BRA /IAL-AD89/2014/41	0.023348
HAdV/SAU/9.15.17	HAdV/CHN/FX1-152772/2015/41	0.031295
HAdV/SAU/9.15.17	HAdV/SAU/SA12680/2014/41	0.031295
HAdV/SAU/9.15.17	HAdV/SWD/GyK253/2000/41	0.031295
HAdV/SAU/9.15.17	HAdV/CHN/D363/2015/41	0.031295
HAdV/SAU/9.15.17	HAdV/USA/4849/2010/41	0.031295
HAdV/SAU/9.15.17	HAdV/TUR/ANKARA10/2016/41	0.031295
HAdV/SAU/9.15.17	HAdV/FRA/MU22/2018/41	0.027311
HAdV/SAU/9.15.17	HAdV/NETH/Tak/2007/41	0.035301
HAdV/SAU/9.15.17	HAdV/BRA/IAL-AD694/2016/40	0.060273
HAdV/SAU/9.15.17	HAdV/CHN/GZ20191121/026/2019/E4_{outgroup}	0.152421

HAdV/SAU/9.15.17	HAdV/BRA/IAL-AD178/2016/41	0.019406
HAdV/SAU/9.15.17	HAdV/IRQ/MU35/2016/41	0.027311
HAdV/SAU/9.15.17	HAdV/IND/BDF/2017/41	0.031295
HAdV/SAU/9.15.17	HAdV/CHN/D240/2015/41	0.031295
HAdV/SAU/9.15.17	HAdV/JPN/SaP3-3F/2012/41	0.031295
HAdV/SAU/9.15.17	HAdV/IND/503214/2009/41	0.031295
HAdV/SAU/9.15.17	HAdv/FIN/HoviX/1979/40	0.068412
HAdV/SAU/9.15.17	HAdV/BRA/IAL-AD09/2016/41	0.023348
HAdV/SAU/9.15.17	HAdV/IND/SWR9/2017/41	0.031295
HAdV/SAU/9.15.17	HAdV/JPN/D27/2011/41	0.031295
HAdV/SAU/9.15.17	HAdV/CHN/NIVD103/2007/41	0.031295
HAdV/SAU/9.15.17	HAdV/SAU/SA12303/2014/40	0.068412
HAdV/SAU/9.15.17	HAdV/JPN/Dugan/2007/40	0.068412
HAdV/SAU/9.15.17	HAdV/CHN/D240/2015/41	0.031295
HAdV/SAU/9.15.17	HAdV/CHN/D4/2011/41	0.031295
HAdV/SAU/9.15.17	HAdV/UK/Dugan/1990/40	0.068412
HAdV/SAU/9.15.17	HAdV/SAU/SA6749/2014/41	0.031295
HAdV/SAU/9.15.17	HAdV/BRA/IAL-AD99/2015/40	0.059793
HAdV/SAU/9.15.17	HAdV/USA/4845/2010/41	0.031295
HAdV/SAU/9.15.17	HAdV/USA/HS12/2009/41	0.031295

The count of base substitutions per site from inter- sequences are displayed. Final dataset included a total of 261 positions. The numbers in red referred to the closest distance (least evolutionary divergence for each sequence. However, the numbers in dark red denoted the next closest distance.

Table S5. Sequences used for phylogenetic analysis of HAdV

Accession number	Sequence nomination	Abbreviated name
MH168374	Human adenovirus sp. strain HAdVF41/IAL-AD89/2014/BRA hexon protein gene	HAdV/BRA/IAL-AD89/2014/41
MK883610	Human mastadenovirus F isolate Human/China/Shanghai/FX1-152772/2015/41	HAdV/CHN/FX1-152772/2015/41
MK962810	Human mastadenovirus F strain SA12680	HAdV/SAU/SA12680/2014/41
KX868523	Human adenovirus 41 isolate GyK253	HAdV/SWD/GyK253/2000/41
KY316163	Human adenovirus 41 isolate SH/2015/D363	HAdV/CHN/D363/2015/41
KF303069	Human adenovirus 41 isolate NY/2010/4849	HAdV/USA/4849/2010/41
MN830351	Human adenovirus sp. isolate 41 ANKARA10/16 hexon gene	HAdV/TUR/ANKARA10/2016/41
MW567966	Human adenovirus 41 isolate MU22/patient E	HAdV/FRA/MU22/2018/41
DQ315364	Human adenovirus 41 isolate Tak	HAdV/NLD/Tak/2007/41
MH223634	Human adenovirus sp. strain HAdVF40/IAL-AD694/2016/BRA hexon protein gene	HAdV/BRA/IAL-AD694/2016/40
MH223623	Human adenovirus sp. strain HAdVF41/IAL-AD178/2016/BRA hexon protein gene	HAdV/BRA/IAL-AD178/2016/41
MG925783	Human adenovirus 41 isolate MU35	HAdV/IRQ/MU35/2016/41
MK028955	Human adenovirus sp. isolate BDF hexon gene	HAdV/IND/BDF/2017/41
KY316162	Human adenovirus 41 isolate SH/2015/D240	HAdV/CHN/D240/2015/41
AB728839	Human adenovirus 41 DNA, complete genome, strain: SaP3-3F	HAdV/JPN/SaP3-3F/2012/41
HQ005289	Human adenovirus 41 strain 503214/KOL/2009 hexon gene	HAdV/IND/503214/2009/41
KU162869	Human adenovirus 40 strain HoviX	HAdV/FIN/HoviX/1979/40
MH223625	Human adenovirus sp. strain HAdVF41/IAL-AD09/2016/BRA hexon protein gene	HAdV/BRA/IAL-AD09/2016/41
MK028954	Human adenovirus sp. isolate SWR9 hexon gene	HAdV/IND/SWR9/2017/41
AB610523	Human adenovirus 41 gene for hexon protein, strain: D27	HAdV/JPN/D27/2011/41
HM565136	Human adenovirus 41 isolate NIVD103	HAdV/CHN/NIVD103/2007/41
MK955319	Human mastadenovirus F strain SA12303	HAdV/SAU/SA12303/2014/40
AB330121	Human adenovirus 40 gene for hexon, strain: Dugan	HAdV/JPN/Dugan/2007/40
AB610520	Human adenovirus 41 gene for hexon protein, strain: D4	HAdV/CHN/D4/2011/41
X51782	Adenovirus type 40 hexon gene	HAdV/UK/Dugan/1990/40
MK962807	Human mastadenovirus F strain SA6749	HAdV/SAU/SA6749/2014/41
MH201117	Human adenovirus sp. strain HAdVF40/IAL-AD99/2015/BRA hexon protein gene	HAdV/BRA/IAL-AD99/2015/40
KF303070	Human adenovirus 41 isolate NY/2010/4845	HAdV/USA/4845/2010/41
JF699082	Human adenovirus 41 Wisconsin isolate HS12 hexon gene	HAdV/USA/HS12/2009/41
MW012924	Human mastadenovirus E isolate HAdV E4 GZ20191121/026 hexon protein gene	HAdV/CHN/ GZ20191121/026/2019/E4 {Outgroup}

Table S6. Best fitting model selection for HAdV using Maximum Likelihood fits of 24 different nucleotide substitution models

Model	#Param	BIC	AICc	lnL	Invariant	Gamma	R	Freq A	Freq T	Freq C	Freq G	A=>T	A=>C	A=>G	T=>A	T=>C	T=>G	C=>A	C=>T	C=>G	G=>A	G=>T	G=>C
**JC**	73	**2138.09**	1613.48	-733.2	n/a	n/a	0.5	0.25	0.25	0.25	0.25	0.08	0.08	0.08	0.08	0.08	0.08	0.08	0.08	0.08	0.08	0.08	0.08
T92	75	2142.60	1603.65	-726.2	n/a	n/a	0.658	0.202	0.202	0.298	0.298	0.06	0.09	0.12	0.06	0.12	0.09	0.06	0.08	0.09	0.08	0.06	0.09
JC+I	74	2142.94	1611.16	-731.0	0.456	n/a	0.5	0.25	0.25	0.25	0.25	0.08	0.08	0.08	0.08	0.08	0.08	0.08	0.08	0.08	0.08	0.08	0.08
JC+G	74	2143.97	1612.19	-731.5	n/a	0.801	0.5	0.25	0.25	0.25	0.25	0.08	0.08	0.08	0.08	0.08	0.08	0.08	0.08	0.08	0.08	0.08	0.08
T92+G	76	2145.96	1599.84	-723.3	n/a	0.808	0.663	0.202	0.202	0.298	0.298	0.06	0.09	0.12	0.06	0.12	0.09	0.06	0.08	0.09	0.08	0.06	0.09
T92+I	76	2146.51	1600.39	-723.6	0.451	n/a	0.663	0.202	0.202	0.298	0.298	0.06	0.09	0.12	0.06	0.12	0.09	0.06	0.08	0.09	0.08	0.06	0.09
K2	74	2147.54	1615.76	-733.3	n/a	n/a	0.652	0.25	0.25	0.25	0.25	0.08	0.08	0.1	0.08	0.1	0.08	0.08	0.1	0.08	0.1	0.08	0.08
K2+G	75	2151.06	1612.11	-730.5	n/a	0.800	0.658	0.25	0.25	0.25	0.25	0.08	0.08	0.1	0.08	0.1	0.08	0.08	0.1	0.08	0.1	0.08	0.08
K2+I	75	2151.30	1612.35	-730.6	0.456	n/a	0.658	0.25	0.25	0.25	0.25	0.08	0.08	0.1	0.08	0.1	0.08	0.08	0.1	0.08	0.1	0.08	0.08
JC+G+I	75	2151.85	1612.89	-730.9	0.000	0.800	0.5	0.25	0.25	0.25	0.25	0.08	0.08	0.08	0.08	0.08	0.08	0.08	0.08	0.08	0.08	0.08	0.08
T92+G+I	77	2155.17	1601.87	-723.3	0.000	0.808	0.663	0.202	0.202	0.298	0.298	0.06	0.09	0.12	0.06	0.12	0.09	0.06	0.08	0.09	0.08	0.06	0.09
HKY	77	2157.61	1604.32	-724.5	n/a	n/a	0.659	0.216	0.189	0.332	0.263	0.06	0.1	0.11	0.06	0.14	0.08	0.06	0.08	0.08	0.09	0.06	0.1
K2+G+I	76	2160.09	1613.97	-730.4	0.000	0.800	0.658	0.25	0.25	0.25	0.25	0.08	0.08	0.1	0.08	0.1	0.08	0.08	0.1	0.08	0.1	0.08	0.08
HKY+G	78	2162.04	1601.57	-722.2	n/a	0.809	0.662	0.216	0.189	0.332	0.263	0.06	0.1	0.11	0.06	0.14	0.08	0.06	0.08	0.08	0.09	0.06	0.1
HKY+I	78	2162.80	1602.33	-722.5	0.451	n/a	0.660	0.216	0.189	0.332	0.263	0.06	0.1	0.11	0.06	0.14	0.08	0.06	0.08	0.08	0.09	0.06	0.1
TN93	78	2167.47	1607.01	-724.9	n/a	n/a	0.662	0.216	0.189	0.332	0.263	0.06	0.1	0.09	0.06	0.15	0.08	0.06	0.09	0.08	0.08	0.06	0.1
HKY+G+I	79	2171.24	1603.60	-722.2	0.000	0.808	0.662	0.216	0.189	0.332	0.263	0.06	0.1	0.11	0.06	0.14	0.08	0.06	0.08	0.08	0.09	0.06	0.1
TN93+I	79	2172.26	1604.63	-722.7	0.443	n/a	0.661	0.216	0.189	0.332	0.263	0.06	0.1	0.1	0.06	0.15	0.08	0.06	0.08	0.08	0.08	0.06	0.1
TN93+G	79	2172.45	1604.81	-722.8	n/a	0.838	0.662	0.216	0.189	0.332	0.263	0.06	0.1	0.1	0.06	0.15	0.08	0.06	0.08	0.08	0.08	0.06	0.1
TN93+G+I	80	2180.24	1605.43	-722.1	0.000	0.837	0.662	0.216	0.189	0.332	0.263	0.06	0.1	0.1	0.06	0.15	0.08	0.06	0.08	0.08	0.08	0.06	0.1
GTR	81	2191.74	1609.77	-723.2	n/a	n/a	0.664	0.216	0.189	0.332	0.263	0.04	0.09	0.09	0.05	0.15	0.13	0.06	0.09	0.06	0.08	0.09	0.08
GTR+G	82	2195.48	1606.34	-720.5	n/a	0.860	0.663	0.216	0.189	0.332	0.263	0.04	0.09	0.1	0.05	0.15	0.13	0.06	0.08	0.06	0.08	0.09	0.08
GTR+I	82	2195.90	1606.77	-720.7	0.433	n/a	0.661	0.216	0.189	0.332	0.263	0.04	0.09	0.1	0.05	0.15	0.13	0.06	0.08	0.06	0.08	0.09	0.08
GTR+G+I	83	2204.70	1608.40	-720.5	0.000	0.863	0.664	0.216	0.189	0.332	0.263	0.04	0.09	0.1	0.05	0.15	0.13	0.06	0.08	0.06	0.08	0.09	0.08

*Models with the lowest BIC scores (Bayesian Information Criterion) are considered to describe the substitution pattern the best (written in red and bold). For each model, AICc value (Akaike Information Criterion, corrected), Maximum Likelihood value (lnL), and the number of parameters (including

branch lengths) are also depicted above. 5 rate categories-discrete Gamma distribution (+*G*) was applied for modeling the evolutionary rates non-uniformity among sites and supposing that a certain fraction of sites is evolutionarily invariable (+*I*). Proposed or estimated values of transition/transversion bias (R) for each model are displayed. They are followed by nucleotide frequencies (*f*) and rates of base substitutions (*r*) for each nucleotide pair.

* Abbreviations: TR: General Time Reversible; HKY: Hasegawa-Kishino-Yano; TN93: Tamura-Nei; T92: Tamura 3-parameter; K2: Kimura 2-parameter; JC: Jukes-Cantor.
