# Supplementary figures and images for: Molecular Epidemiology and Surveillance of Human Adenovirus and Rotavirus A Associated Gastroenteritis in Riyadh, Saudi Arabia
Source: Trop Med Infect Dis. 2023 May 15;8(5):279. doi: 10.3390/tropicalmed8050279 (PMC10221376; doi:10.3390/tropicalmed8050279)

## Supplementary Materials:

RComb-F: CCACAAYTDTATTGTGATTA

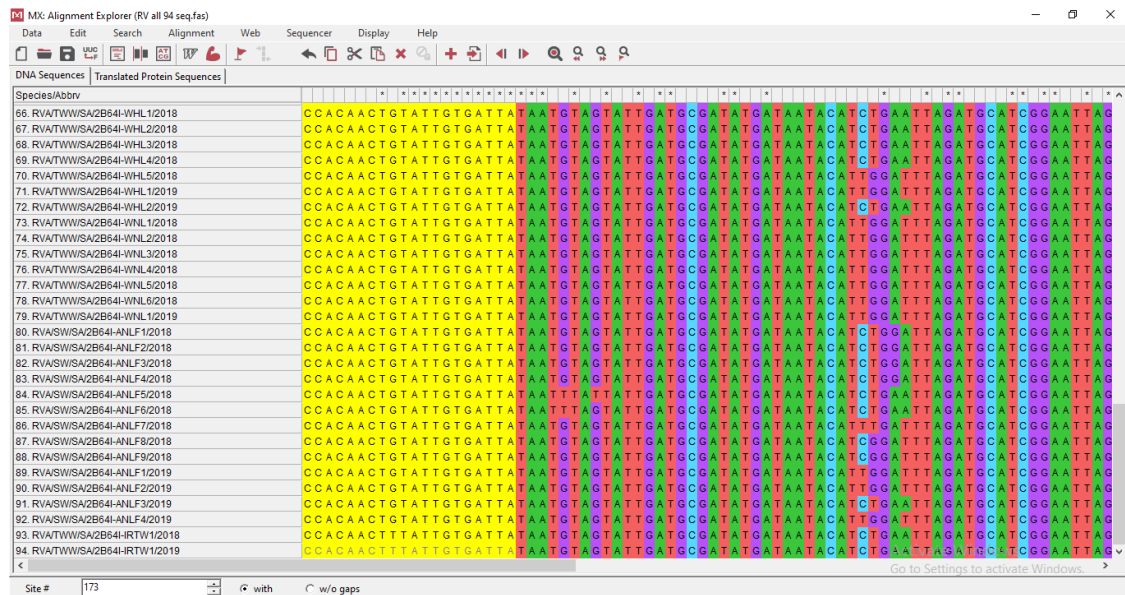

**RComb-R: CCCATYGATATCCAYTTATT**

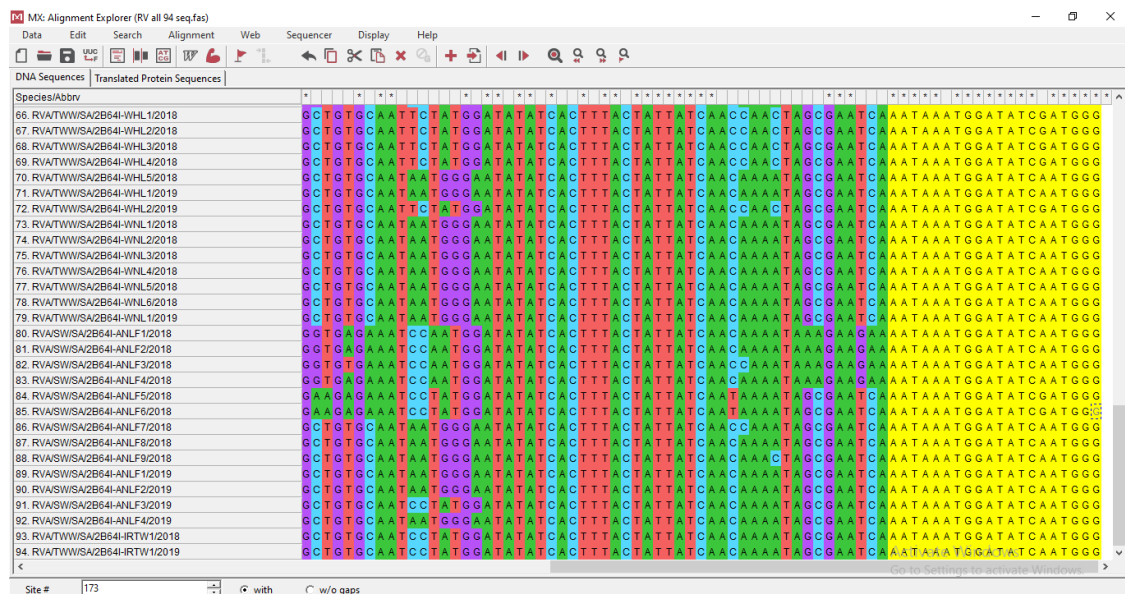

Supplement: Supplementary file 1 [file tropicalmed-08-00279-s001.zip › Rota primers positions (RComb).pdf]
